# Side-Stream Based Marine Solubles From Atlantic Cod (*Gadus morhua*) Modulate Appetite and Dietary Nutrient Utilization in Atlantic Salmon (*Salmo salar L*.) and can Replace Fish Meal

**DOI:** 10.1155/anu/4872889

**Published:** 2025-01-30

**Authors:** Hanne Jorun Sixten, Ivar Rønnestad, André S. Bogevik, Tone Aspevik, Åge Oterhals, Ana S. Gomes, Floriana Lai, Ingvill Tolås, Virginie Gelebard, Marie Hillestad, Katerina Kousoulaki

**Affiliations:** ^1^Department of Research and Development, BioMar AS, Trondheim, Norway; ^2^Department of Biological Sciences, University of Bergen, Bergen, Norway; ^3^Department of Nutrition and Feed Technology, NOFIMA, Fyllingsdalen, Norway; ^4^Institute of Marine Research, Tromsø, Norway; ^5^Department of Biological Sciences, NTNU Ålesund, Ålesund, Norway

## Abstract

Whitefish fisheries' side-stream biomass is an abundant underutilized resource that can be valorized to benefit future aquaculture sustainability. Four novel ingredients based on side-streams from Atlantic cod (*Gadus morhua*) fileting were produced. FM-hb, a fish meal (FM), and FPH-hb, a fish protein hydrolysate based on heads (h) and backbones (b); FM-hbg, a FM based on heads, backbones, and viscera/guts (g); and FPC-g, a fish protein concentrate based on viscera preserved in formic acid. Four diets were prepared containing one of the ingredients replacing 50% of the dietary FM protein, in addition to a positive (FM10) and a negative (FM5) control. The six diets were fed to triplicate tanks with Atlantic salmon (*Salmo salar* L.; 113 ± 1 g) over 8 weeks. Besides general performance, gut and brain gene expression for selected hormones and key neuropeptides involved in the control of appetite and digestive processes were studied during feeding and postprandial, and possible reference levels for Atlantic salmon were established. All side-stream-added diets performed well, with no significant differences in performance and biometrics between the treatments. Some gene expression differences were observed, but no well-defined patterns emerged supporting clear dietary effects related to digestive performance or appetite. However, in the brain, a short-time upregulation of *agouti-related protein-1* (*agrp1*), corresponded to higher cumulative feed intake (FI) for the FM10 diet supporting notions that this may be a candidate biomarker for appetite in salmon. Expression of stomach *ghrelin-1* (*ghrl1*) was higher than *ghrelin-2* (*ghrl2*) and *membrane-bound O-acyltransferase domain-containing* 4 (*mboat4*), and midgut *peptide YYa-2* (*pyya2*) and *glucagon-a* (*gcga*) were higher than *peptide YYb-1* (*pyyb1*). A comparison showed that midgut *peptide YYa-1* (*pyya1*), *pyya2*, and *gcga* expressions were higher than in the hindgut, which is opposite of what is found in mammals. In conclusion, this study shows that sustainable side-stream raw materials with different characteristics can partly replace high-quality commercial FMs giving similar performance.

## 1. Introduction

Mariculture is more climate-friendly than land-based meat production and represents an important contribution to a more sustainable way to feed the world healthy animal proteins and fats [1]. The aquaculture industry has shown high growth rates since the 1980s and 90s reaching an annual global production of aquatic animal production of 94.4 million tons in 2022, while the world capture fisheries have remained stable [2]. In the last decade, marine ingredients have accounted for a declining share, while plant raw materials show an increasing inclusion in feeds for Atlantic salmon (*Salmo salar* L.) [3, 4]. Marine side-stream products represent a largely underutilized marine biomass that can provide high-quality nutrients in relevant volumes and contribute to increased growth and sustainability of the aquaculture industry [2, 5]. The use of such marine raw materials will reduce the dependency on traditional fish meal (FM) and fish oils, in addition to plant ingredients claiming large land areas that cause deforestation and suppress the production of plants for human consumption [3]. In Norway, the pelagic catch volumes (herring, mackerel, capelin, sandeel, pout, and blue whiting) are fully utilized, both for direct human consumption and to produce FM and fish oil, and side-stream products from freshly landed fish processed as silage/fish protein concentrate (FPC) [6, 7]. However, whitefish side-stream products (53% from Atlantic cod, the rest from haddock, pollock, halibut, ling, tusk, redfish, and wolffish) from offshore catch in the Norwegian Sea and Barents Sea are still not fully utilized mainly due to the distance between the slaughter and processing sites and the lack of investment in onboard equipment for utilization of whitefish byproducts processed into FM, FPC, or fish protein hydrolysates (FPHs) [6, 8]. Using these residuals after fish processing as conventional FM and fish oil replacements in fish feeds may contribute to the reduction of fish-in fish-out ratio (FIFO) below 1 and reduced ASC feed standards for FM, forage fish dependency ratios (FFDRs) for FM and fish oil, and offer large economic, nutritional, and environmental values [1, 3, 9].

Fish and seafood are good sources of bioactive, functional, and nutritional components giving the feed more favorable, safe, and healthy combinations of proteins, amino acids (AAs), fats, fatty acids, phospholipids, minerals, trace elements, and key vitamins with positive effects in fish acceptance, performance, and health [1]. Processing of fish by endogenous or added enzymes produces FPC and FPH high in water-soluble proteins (WSPs), and stickwater (SW) produced after removal of solids and oil and added back in the FM production are also high in WSPs [10, 11]. WSPs from marine sources, with high contents of appetite-stimulating components, such as low molecular components and free AA (FAA), induce an increase in feed intake (FI) in Atlantic salmon, in addition to better utilization and growth [11–15]. Marine extracts or hydrolysates are commonly used in plant-based fish feeds for aquatic organisms to balance composition of essential AAs (EAAs), fatty acids, and phospholipids and to make the feeds more palatable, stimulating appetite, FI, and growth performance [11, 16–22], but can also improve physical pellet quality (PPQ) and structure of the feed pellets [23–26].

Fish primarily detect food through smell and sight; moreover, the texture and taste of the diet are key factors for whether food will be swallowed or rejected by the fish [27–29]. The intake of feed during a meal results from a shift from hunger to satiety, and the appetite and FI are controlled by complex signaling pathways between the brain and peripheral tissues and organs like the gastrointestinal tract (GIT), liver, and adipose tissue, among others [30, 31]. The hypothalamus (HYP) acts as the neural hub for controlling appetite and energy balance by integrating peripheral afferent hormonal and neural signals including those from GIT, which plays a crucial role in the gut–brain axis communication [32, 33]. In the HYP, there are neural networks that express neuropeptides that either stimulate appetite/hunger (orexigenic signals), such as *neuropeptide Y* (Npy) and *agouti-related protein* (Agrp), or stimulate satiety/inhibit appetite (anorexigenic signals), such as cocaine- and *amphetamine-regulated transcript* (Cart) and *proopiomelanocortin* (Pomc) [33]. An increasing number of peptides homologous to the mammalian appetite-controlling hormones have been isolated and characterized in fish, including Atlantic salmon [33, 34]. Recent studies have identified the distribution of different neuropeptide expressions across different regions of the brain [35] and revealed several new paralogs compared to some of the genes in the Atlantic salmon genome. That is two *agrp* paralogs named *agrp1* and *agrp2* [36, 37]; three *pomc* paralogs named *pomca1*, *pomca2*, and *pomcb* [38, 39]; three *npy* paralogs named *npya1*, *npya2*, and *npyb* [40]; and 10 *cart* paralogs named *cart1a*, *1b1*, *1b2*, *2a*, *2b1*, *2b2*, *3a1*, *3a2*, *3b*, and *4* [41, 42]. The GIT is a multifunctional endocrine organ that serves many physiological functions, producing a large range of signaling peptides and hormones acting on several tissues [43]. Signals in the GIT originate in enteroendocrine cells (EECs) located in the epithelium facing the lumen containing species-specific nutrient receptors for carbohydrates, fat, and AAs [32, 33]. In mammals as well as fishes, there is a range of specific hormones produced in the GIT which has a primary effect in regulating the digestive process, but also affects appetite and feeding behavior. This includes the appetite-stimulating factors (orexigenic), like *ghrelin* (Ghrl) and its activator *ghrelin O-acyltransferase or membrane-bound O-acyltransferase domain-containing 4* (Goat or Mboat4) [44], or appetite-inhibiting (anorexigenic) factors, like *peptide YY* (Pyy), *cholecystokinin* (Ckk), *glucagon* (Gcg), *glucagon-like peptide 1* and *2* (Glp-1 and Glp2), *insulin*, and *leptin* [31, 33, 45]. There are also several gene paralogs for appetite related hormones in the GIT in the Atlantic salmon genome, with two *ghrl* paralogs named *ghrl1* and *ghrl2*; two *cck* paralogs named *cck-l* and *cck-n*; and *pyy* [46]. In this paper, paralog *agrp1*, as well as *npy* and *cart* were studied in the whole brain of Atlantic salmon, using the primers pair in the original description of these genes. Thus, the analysis used in this study does not discriminate the *npy* or *cart* paralogs as they were not available at the time of analysis. In the stomach, paralogs *ghrl1* and *ghrl2*, as well as *mboat4* were studied. While in midgut and hindgut, the paralogs of *pyy* and *gcg*, named *peptide YYa-1* (*pyya1*), *peptide YYa-2* (*pyya2*), *peptide YYb-1* (*pyyb1*), *peptide* YYb-2 (*pyyb2*), and *glucagon-a* (*gcga*) were described for the first time in Atlantic salmon in the present study.

The fish were conditioned to a frequent feeding regime (2–3 min eight times each day) with six different diets, and the effects of the diets were investigated after feeding for 21 and 56 days as well as during feeding and postprandially. Changing the composition of marine raw materials and their inclusion level in fish feed requires good knowledge of food safety, ingredients' nutritional value, and technical characteristics. In the present study, we examined the effect of replacing 50% of FM protein by different combinations (heads, backbones or/and viscera/guts) or differently processed (FM, hydrolysate, or silage) cod residual ingredients in low-FM feeds on Atlantic salmon appetite and performance. The main objective was to investigate the performance of novel marine raw materials based on side-stream products compared to commercial FMs, to possibly support increased sustainability and increased value creation in aquaculture, and to assess to what extent these materials affected intrinsic factors regulating appetite, FI, and metabolism [47]. An important aspect was to explore if increased use of marine side-stream materials may improve precision nutrition and lead to a more sustainable use of the raw materials. This may help feed producers and farmers develop improved tools for optimization of farming.

## 2. Material and Methods

### 2.1. Side-Stream Resources and Processing

Side-stream fractions consisting of heads, backbones, and viscera/guts, the latter comprising visceral organs, that is gut with surrounding tissues and organs, including liver and heart, were collected after filleting of Atlantic cod (*Gadus morhua*) harvested during autumn in the Finnmark region (Norway). The head and backbone fractions were frozen at −20°C immediately after the filleting operation, while formic acid was added to the viscera fraction for a duration of 4 weeks to produce a stable silage at pH < 4 and 8−10°C, prior to shipment to Nofima Aquafeed Technology Centre in Bergen (Norway). In total, four ingredients (1–4) were produced based on different fractions from thawed and ground materials and mixed using thermal processing (i.e., FM process) and enzymatic hydrolysis, in addition to silage. To produce the FM FM-hbg (1) cod heads, backbones, and viscera were mixed at a ratio of 20:20:60, ground, cooked (<85°C, 20 min), and mechanically dewatered in a double screw press (Stord Bartz, Norway). The press liquid was separated into oil and SW phases in a separator (GEA Westfalia Separator, Oelde, Germany). SW was concentrated in a four-stage evaporator, mixed into predried press cake (PC), and dried to final dry matter (DM) in a hot air Forberg dryer (Forberg, Oslo, Norway). Producing FM FM-hb (2) cod heads and backbones were mixed 50:50 and subjected to thermal processing following the same procedure as for FM-hbg. Cod FPH-hb (3) was produced from heads and backbones mixed 50:50, added water (1:1), and heated to 55°C in a 200 L jacketed stirred tank reactor. A commercial blend of microbial endopeptidases (Protamex, 0.15% w/w, Univar, Norway) was added to the slurry and the hydrolysis ran for 50 min before thermal inactivation at 90°C for 10 min. Bones and nonsolubilized matter were separated in a Jesma VS 20/65 Roto-Fluid sieve (Jesma, Velje, Denmark) with a 100 µm sieve net opening. The mixed bone and sludge phases were dried in a hot air tray dryer, whereas the hydrolysate phase was concentrated and spray dried. The solid phase was milled and mixed with the spray-dried hydrolysate to a final meal. The cod FPC-g (4) was based on the silage product from fish viscera (100%) fraction. The crude silage concentrate was cooked and suspended solids were removed in a Jesma sieve (Jesma-Matodor AS, Vejle, Denmark) before separation into oil and liquid phases in a decanter centrifuge (Flottweg Tricanter Z-23-3; Vilsbiburg, Germany). The separated silage was concentrated in a four-stage evaporator (APV Anhydro; København, DK) to a stable FPC.

### 2.2. Feed Formulation and Production

Six feeds were produced at BioMar Tech Center (Brande, Denmark) using the novel feed ingredients produced (1–4); a control feed with a medium content of FM (FM10), a control feed with low (5%) FM level (FM5), and four low FM diets where 5% FM (compared to FM10) were replaced by 4.9%–5.5% experimental ingredients (FM-hbg, FM-hb, and FPH-hb), or 14% inclusion of FPC-g due to lower DM content. On a DM basis, each novel ingredient substituted 50% of the dietary FM protein, thereby, contributing precisely 3.42% marine-derived protein (Table 1). There was a slight difference between the diets on vegetable protein inclusion levels. However, all feeds were balanced for minerals, vitamins, AAs, and fatty acids according to the known nutritional requirements of Atlantic salmon and in-house experience at BioMar AS (Table 2). The dry feed batches were homogenized, preconditioned in an atmospheric conditioner (Clextral, Firminy, France), and subsequently processed in a twin-screw extruder (BC 45; Clextral). Postextrusion, the extrudates were dried in a six-layer column dryer (Geelen type). The dried pellets were coated with oil mix in a BES vacuum coater (Brande Entreprenør Service). After drying and cooling, the 4.5 mm diets were packed in 25-kg plastic bags, and stored at 4°C for further analysis and growth experiment.

### 2.3. Fish Experiment and Sampling

For the feeding trial of this study, we used 1800 Atlantic salmon (*S. salar* L.) postsmolts produced by Nofima (BO1-17; Sunndalsøra, Norway), with an average start body weight of 113 ± 1 g. The trials were conducted in a seawater flow-through system at Nofima's Research Station for Sustainable Aquaculture (Sunndalsøra, Norway). Prior to the trial the fish were fed Nutra Olympic, 3 mm (Skretting). At the trial start, the experimental fish were distributed into 18 indoor tanks (1 m^2^, 56 cm water depth). Each of the six feeds were randomly assigned to triplicate tanks for 8 weeks, and feeding was performed for 2–3 min eight times each day to reduce hierarchy in the tanks and with 20% overfeeding based on the recorded daily feed consumption amounts per tank [48]. The average water temperature during the trial was 10.3 ± 0.8°C, with a 24-h light photoperiod. The fish were sampled on day 21 (Sampling 1) and day 56 (Sampling 2). In each tank, three fish were sampled per sampling point either during feeding (feeding) or 1–2 h after the last meal (postprandial) with 2 days between samplings, so that the remaining fish could recover from “sampling-induced stress.” The sampled fish were euthanized using a lethal dose of MS-222 (3 g/L tricaine methanesulfonate, Scan-Vacc, Hvam, Norway). Standard length, body weight, liver weight, heart weight, and gutted weight were recorded for the calculation of Fulton's condition factor (CF) [49], dress-out percentage (D%), hepatosomatic index (HSI), cardio somatic index (CSI), and viscera somatic index (VSI). Brain, stomach, midgut, and hindgut were carefully dissected, stored in RNAlater (Thermo Fischer Scientific, Waltham, MA, United States) at 4°C for 24 h, and subsequently stored at −80°C until further gene expression analyses. On day 46 and 47, 44–47 fish were removed from each tank and measured for body weight, to reduce the tank biomass. After a couple of days with final sampling, the remaining fish in the tanks were weighed and stripped for feces to calculate the apparent digestibility coefficient (ADC) of dietary nutrients.

### 2.4. Chemical Analysis

Chemical analyses were carried out at the accredited laboratory of Nofima, Biolab (Bergen, Norway). Crude protein of feeds and samples of GIT content were analyzed by the Kjeldahl method (*N* × 6.25; ISO 5983-1997). Moisture (ISO 6496-1999) and ash (ISO 5984-2002) were determined gravimetrically after drying preweighed samples in porcelain cups for 4.5 h at 103 ± 1°C, followed by incineration of the dried samples at 550°C ± 20°C for 16 h. Total lipid in diets and fish samples was quantified [50], followed by quantification of fatty acid in GC and lipid classes in HPLC-CAD. Yttrium was determined by inductively coupled plasma atomic emission spectroscopy (ISO 11885-1996). The WSP fraction was extracted with boiling water, the extract was then filtered using a paper filter, and the crude protein content in the water phase was determined by the Kjeldahl method. Molecular weight (MW) distribution analysis in the water-soluble fraction was done by size exclusion chromatography (SEC; 1260 series HPLC Agilent Technologies) with a Superdex Peptide 10/300GL column (GE Healthcare, Uppsala, Sweden), acetonitrile with TFA as eluent, and UV detection at 190−600 nm [51]. The samples were solubilized in water containing 3 g/kg sodium dodecyl sulfate, centrifuged for 10 min at 1710.5 × *g*, decanted, and filtered before applied to the column. The following components were used to define the standard curve to relate MW of peptides to elution time: carbonic anhydrase (MW: 29000), lysozyme (MW: 14300 Da), Cyt C (MW: 12400), aprotinin (MW: 6500), alberta 4 (MW: 3249.38), insulin A (MW: 2531.64), alberta 3 (MW: 2441.54), gastrin I (MW: 2126.28), alberta 2 (MW: 1633.7), polymyxin (MW: 1470), substrate P (MW: 1347.63), [Val 4]-Ang III (MW: 917.06), alberta 1 (MW: 825.86), (Leu)3 (MW: 357.49), and Gly (MW: 75.07).

### 2.5. Micro-CT Scan

Micro-CT scanning of feed pellets was performed using a SkyScan 1275 X-ray microtomography (Bruker MicroCT, Kontich, Belgium), and the scan parameters were optimized and adjusted (*n* = 3 per diet); the scans were done with no filter, a source voltage of 20 keV, and the source current at 175 uA. The scans were high resolution, with an Image Pixel Size of 7 μm, a 360° rotation, a frame averaging 2, and rotation steps of 0.4°. NRecon (v 1.7.3.1 Bruker MicroCT, Kontich, Belgium) was used to reconstruct the scans; the smoothing was set to 0, the beam hardening correction to 45%, and the ring artifact reduction to 8. The CT analyzer (CTAn 1.20.3.0, Bruker MicroCT, Kontich, Belgium) was used to choose the pellet as a volume of interest (VOI). For each picture the lower gray threshold was adjusted between 100 and 140 to find true porosity as the pellets were coated with oil; that is, separation between pellet structure versus open pores plus pores filled with oil and percent object volume (OV/VOI) and total porosity was calculated per picture. The structure thickness distribution (µm) versus volume (%) was also calculated.

### 2.6. Gene Expression Analysis

Total RNA from the whole brain was extracted and DNase treated using the QIAsymphony RNA kit (Qiagen, Hilden Germany) in the QIAsymphony SP automatic system (Qiagen, Hilden, Germany), following the manufacturer's protocol. A NanoDrop ND-1000 spectrophotometer (Thermo Fisher Scientific, MA, USA) and a 2100 Bioanalyser with RNA 6000 nano Kit (Agilent Technologies, CA, USA) were used to assess the quantity, quality, and integrity of the extracted total RNA. Complementary DNA was synthesized from 2.0 μg of total RNA using oligo (dT_20_) primer and the Superscript III kit (Thermo Fisher Scientific) using a Microlab STAR Liquid Handling system (Hamilton Robotics, Reno, USA), following the manufacturer's instructions. For the whole brain, stomach, midgut, and hindgut tissues, total RNA was extracted using TRI reagent (Sigma-Aldrich, MO, USA), following the manufacturer's protocol. To avoid any remnants of genomic DNA, 10 µg of total RNA was treated with TURBO DNase-free Kit (Ambion Applied Biosystem, CA, USA) with 1 µl of DNase (2 Unit/µl) in a 30 µl reaction final volume. A NanoDrop ND-1000 spectrophotometer (Thermo Fisher Scientific, MA, USA) and a 2100 Bioanalyser with RNA 6000 nano Kit (Agilent Technologies, CA, USA) were used to assess the quantity, quality, and integrity of the extracted total RNA, respectively. First strand cDNA was synthesized from 1.2 µg (brain, stomach, midgut, and hindgut) of the total RNA sample using SuperScript III Reverse Transcriptase (Invitrogen, CA, USA) and oligo (dT)_20_ primers in a total reaction volume of 20 µl. The mRNA expression of *npy*, *agrp1*, and *cart* in the brain; *ghrl-1*, *ghrl-2*, and *mboat4* in the stomach; and *pyy* (*a1*, *a2*, *b1* and *b2*) and *gcga* in the midgut and hindgut and was quantified by real-time quantitative RT-PCR (qPCR) using Atlantic salmon gene-specific primers (Table 3). For a*grp1*, *npy*, *cart*, *ghrl-1*, *ghrl-2*, and *mboat4* mRNA expression analysis were performed using published qPCR primers [36, 38, 42, 52, 53]. For *pyy* and *gcga* genes, specific qPCR primers were designed based on the sequences retrieved from the Atlantic salmon genome databases available in GenBank. Primers were analyzed for quantitation cycle (Cq), efficiency, and melting peaks. All qPCR products were resolved on a 2% agarose gel, purified using QIAquick Gel Extraction Kit (Qiagen, Hilden, Germany), and cloned into a pCR4-TOPO vector (Thermofisher Scientific, Massachusetts, USA). Sequencing was performed at the University of Bergen Sequencing Facility (Bergen, Norway) and their identity was confirmed using blastn analysis against the Atlantic salmon genome database. qPCR reactions were performed in duplicate using iTaq Universal SYBR Green Supermix (Bio-Rad, CA, USA) in a 20 μl final reaction volume. The qPCR reactions were performed in a Bio-Rad CFX96 Real-Time System with the following cycling conditions: 95°C for 30 s, 40 cycles of 95°C for 5 s, and 60°C for 25 s. Melting curve analysis over a range of 65–95°C (increment of 0.5°C for 2 s) allowed for the detection of possible nonspecific products and/or primer dimers. To quantify the absolute mRNA abundance for each target gene, PCR products were used to generate a standard curve using a tenfold dilution series [38]. For *agrp1*, *npy*, *cart*, *ghrl1*, *ghrl2*, *mboat4*, *pyya1*, *pyya2*, *pyyb1*, *pyyb2*, and *gcga*, standard curves per were generated from the target gene cloned into pCR4-TOPO vector (Thermo Fisher Scientific), using a tenfold stepwise dilution series. The copy number was determined for each gene/sample based on the respective standard curve, using the following equation:

  
$$\begin{array}{c} \text{Copy number}=10^{\left(\frac{C_{q}-\text{Intercept}}{\text{Slope}}\right)}. \end{array}$$

The copy number was expressed as absolute numbers and not normalized to the total ng of RNA. Sequence of specific primers used for reverse transcriptase quantitative PCR (RT-qPCR) analysis is shown in Table 3.

### 2.7. Statistics

Fish performance and gene expression data were analyzed in R version 4.3.0, using the tidyverse and emmeans packages [54–56]. Generalized linear mixed modeling (GLMM) with gamma distribution and log link function was used to model the gene expression data as a function of diet, nutritional status, and sampling, including an interaction term between these three factors. For fish body weight a GLMM with a gamma distribution and log link function was applied to model the data in function of diet and time, including an interaction between these two variables. For all GLMMs, tank was included as a random effect to account for possible tank-to-tank variation. For fish performance and biometrics data, linear modeling (LM) assuming a gaussian distribution was applied to model the data as a function of diet. LM was also used for correlation analysis between performance and biometrics data. After the GLMM and LM analyses, a Tukey's post hoc pairwise tests were conducted to estimate conditional contrasts between groups using the emmeans function. Effects were considered significant when *p* < 0.05.

## 3. Results

### 3.1. Raw Material Characteristics

The protein content was highest in the hydrolysate FPH-hb (70%), followed by the FM products (FM-hbg and FM-hb at 62%–63%), and lowest in the silage FPC-g (25%) due to a high content of water from the process and fat from visceral part of the fish (Table 4). The latter was also reflected in a higher fat content in FM-hbg. Similarly, the presence of head and backbones increased the ash and phosphorous (P) content in FM-hb, FM-hbg, and FPH-hb compared to FPC-g. WSP content was highest in the FPH-hb (50% of the ingredient, as is), followed by FM-hbg (31%), and was approximately similar between FM-hb and FPC-g (21%–23%; Table 4). Nevertheless, the FPC-g had a higher content of small peptides <1000 Da (89% of soluble protein), also reflected in the FM-hbg (52%), both with high level of FAA (36% and 32% of soluble protein, respectively), while the FPH-hb had a high content both of smaller <1000 Da (47%) and intermediate peptide sizes 1000–6000 Da (47%), and the soluble fraction of the FM-hb ingredient had larger peptides like >20.000 Da (42%) and intermediate/large peptides 6000–20,000 Da (31%). Minor differences in fatty acids were observed, while viscera inclusion appeared to lower phospholipid content in the ingredients.

### 3.2. Feed Characteristics

All feeds were balanced to have the same content of protein, fat, and energy, as well as EAAs, FA, and other nutrients according to Atlantic salmon requirements. As a result, the WSP content was higher in FPH-hb and FPC-g diets (10 g/100 g) compared to FM10, FM-hbg, and FM-hb diets (8 g/100 g), but at the same level in the FM5 diet (10 g/100 g) due to higher inclusion of crystalline AAs (Table 5). P was slightly higher in the diets with head and bone ingredients.

Differences were also observed in pellet quality, where the negative control (FM5) had significantly higher pellet hardness (Kahl, 44 N) than FM-hbg, FPH-hb, and FPC-g (30–35 N), and a trend to higher Kahl than FM10 and FM-hb (39 N; Table 6). FPC-g had significantly lower Kahl compared to FM10 and FM-hb. FM5 had a significantly lower water stability index (WSI, 61%) compared to the rest of the diets (68%–75%), and FPC-g had significantly lower WSI (68%) compared to FM10 and trend to lower WSI compared to FPH-hb (74%–75%). The feeds had sufficient water stability not to dissolve in the feed spill collection system, thus, FI could be measured for all feeds. Micro-CT scans of the pellets showed a difference in total porosity (%) and structure separation distribution (µm) between the diets. FPH-hb had significantly more pore volume (total porosity, 44%) compared to FM-hbg and FM-hb (33%–36%).

### 3.3. Fish Performance

The fish mean body weight per tank increased significantly from 113 ± 1 g (number of fish weighed (*n* = 100) at the start of the trial) to 170 ± 20 g (*n* = 100) at mid sampling on day 21, to 228 ± 10 g (*n* = 44–47 fish weighed and removed to reduce biomass in the tanks) on day 47, and to 269 ± 10 g (*n* = 45–47) at end sampling and termination of the experiment on Day 56. Salmon fed the FM10 and FM-hb diets had the highest final weight (274 g), while those on the FM5 had the lowest final weight (264 g). There were, however, no significant differences (*p* > 0.05) in intermediate or final mean body weights between dietary treatments (Table 7). Like body weight measurements, the specific growth rate (SGR) and thermal growth coefficient (TGC) [57] showed no significant dietary effects (*p* > 0.05), nevertheless higher values in FM10 and FM-hb (SGR: 1.60–1.61) compared to the rest of the diets (1.53–1.57, Table 7). Additionally, FI in the experimental periods showed no significant dietary effects, although salmon fed FM10 and FPC-g had slightly higher intake (1.26–1.30) compared to the other groups (1.16–1.20). This resulted in a higher feed conversion ratio (FCR) in the FM10 and FPC-g groups (0.72–0.73) compared to the others (0.66–0.68; Table 7).

### 3.4. Apparent Digestibility

There were no differences in digestibility of lipid, protein, and energy between the dietary groups. ADC values for FPH-hb were removed due to an error in feed yttrium values, caused by a dosing mistake. Salmon in the FM-hb treatment showed a significantly lower ADC of P compared to FM10 and FM5 (*p*  < 0.05; Table 8). FPC-g treatment showed significantly lower ADC of glutamic acid, methionine, isoleucine, and valine compared to FM5, in addition to lower ADC of valine compared FM10. FM5 showed significantly higher ADC of lysine value compared to the rest of the diets.

### 3.5. Body Composition

The analyses of body composition showed only minor differences between Atlantic salmon sampled on day 21 and 56, or between dietary treatments (Table S6), while feeding and postprandial fish were merged for the fish biometric calculations. The resulting average values (*n* = 72) are for CF 1.20 ± 0.06, D% 88.9 ± 0.9, VSI 9.84 ± 0.86, HSI 1.14 ± 0.10, CSI 0.14 ± 0.02, and gallbladder percentage (GB%) 0.09 ± 0.03. Even though not significant, there was a lower D% and higher VSI in FM10 group compared to salmon fed the other feeds, and HSI was higher in FM10 compared to the rest (Table 9). There were no significant dietary differences in total fat content in muscle 23.2 ± 2.8, liver 5.0 ± 0.5, or viscera 9.8 ± 0.5 of salmon sampled on day 56 (Table 9), nor in relative retention (RT%) of fat in muscle, viscera, or liver (results not included).

### 3.6. Gene Expression

**Figure 1:**
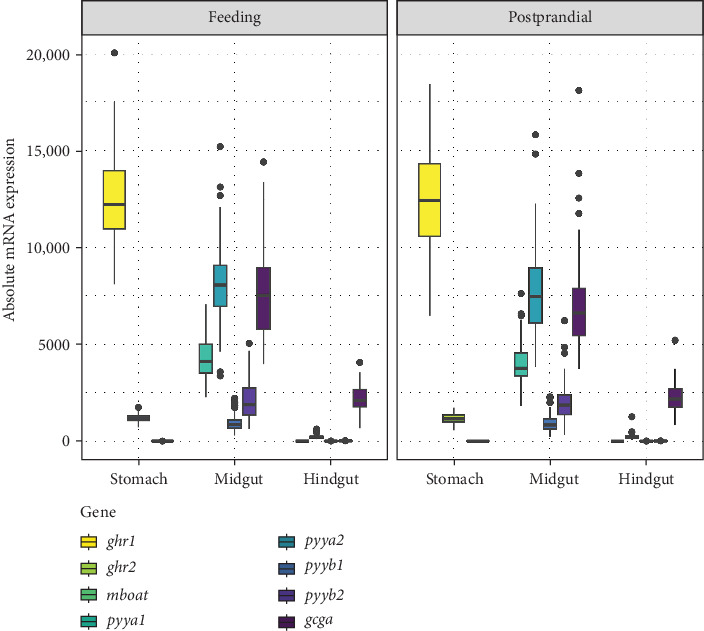
Comparison of the absolute mRNA expression in gastrointestinal tract (GIT); stomach (*ghrelin*-1 (*ghrl1*), *ghrelin*-2 (*ghrl2*), *membrane-bound O-acyltransferase domain-containing 4* (*mboat4*)); midgut; and hindgut (*peptide YYa*-1 (*pyya1*), *peptide YYa*-2 (*pyya2*), *peptide YYb*-1 (*pyyb1*), *peptide YYb*-2 (*pyyb2*), and *glucagon-a* (*gcga*)) dependent on status feeding and postprandial and independent of diet (d) or sampling time. Boxplot shows the median (solid horizontal line), the first and third quartiles (lower and upper limit of the box), the whiskers indicate the 1.5 × interquartile range, and dots are data points outside of the defined range. For detailed statistically significant information, see Tables S7–S10 and Figures S3–S6.

Comparison of the absolute mRNA expression in brain, stomach, midgut, and hindgut showed that the expression varied largely between the different tissues, genes, and paralogs in this study. Gene expression of *ghrl1* in the stomach was highest followed by *pyya2*, *gcga*, and *pyya1* in the midgut. In general, there was a similar mRNA expression level of the genes in fish with status feeding and postprandial, and all *pyy* paralogs and *gcga* were expressed higher in the midgut compared to the hindgut (Figure 1).

#### 3.6.1. Brain

**Figure 2:**
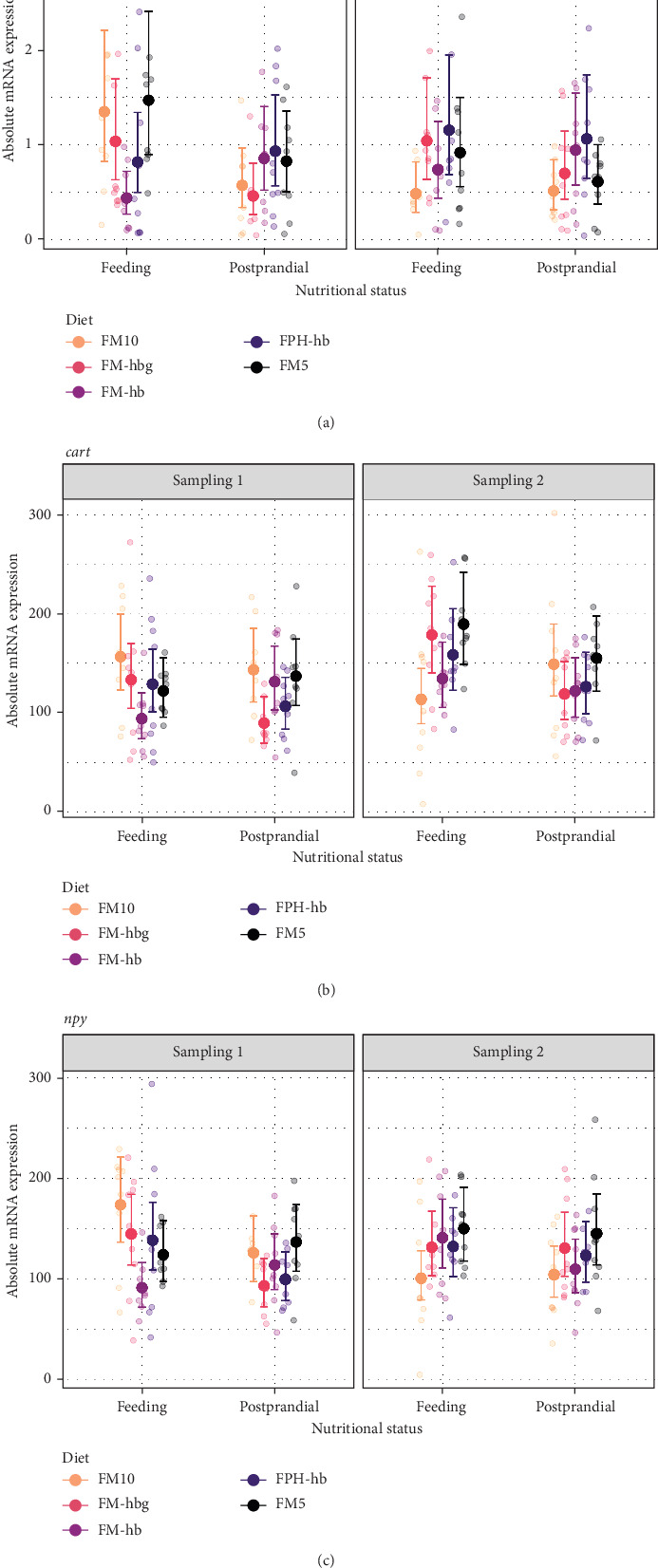
Model prediction of values and raw data of absolute mRNA expression of (a) *agouti-related protein*-1 (*agrp*1), (b) *cocaine- and amphetamine-regulated transcript* (*cart*), and (c) *neuropeptide Y* (*npy*) in Atlantic salmon whole brain (sample size per group = 9). The dots indicate model-predicted means and 95% confidence intervals, respectively, for each diet (d) per sampling (Sampling 1 and Sampling 2) and nutritional status (feeding and postprandial). For detailed statistically significant information, see Table S7 and Figure S7.

The overall level of mRNA expression for *agrp1*, *npy*, and *cart* was 0.9 ± 0.7 (*n* = 174), 126 ± 47 (*n* = 177), and 135 ± 52 (*n* = 177), respectively, in the whole brain. Independent of status or sampling time, the three genes exhibited similar expression patterns between the diets, especially, *npy* and *cart*. The *agrp1* gene exhibited some distinct differences. In the feeding group, there was a significantly (*p*  < 0.05) higher expression of *agrp1* for FM10 at day 21 compared to day 56 (Figure 2). There was an effect of nutritional status on day 21 as feeding fish had significantly higher expression of *agrp1* than postprandial fish for FM10 and FM-hbg. A dietary effect was also observed in feeding fish on day 21, where there was a significantly higher expression of *agrp1* in the FM10 and FM5 as compared to the FM-hb treatment.

#### 3.6.2. Stomach

**Figure 3:**
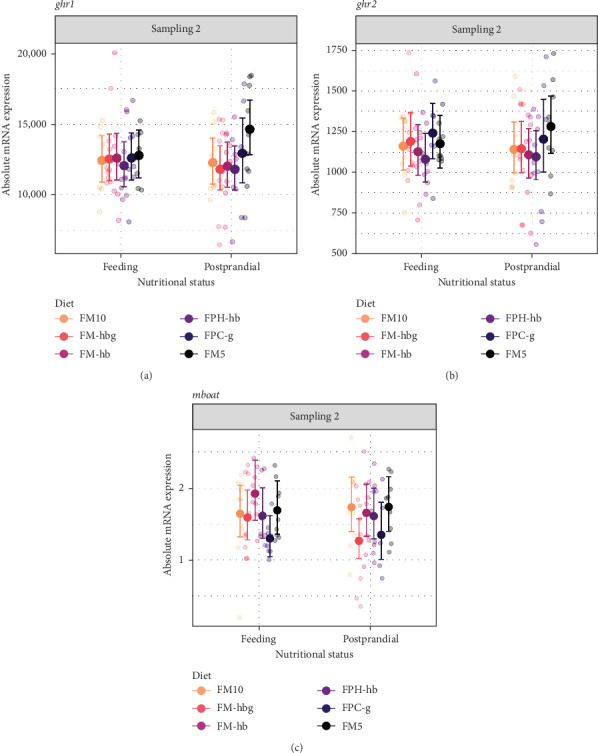
Model prediction of values and raw data of absolute mRNA expression of (a) *ghrelin*-1 (*ghrl1*), (b) *ghrelin*-2 (*ghrl2*), and (c) *membrane-bound O-acyltransferase domain-containing 4* (*mboat4*) in Atlantic salmon stomach (sample size per group = 9). The dots indicate model-predicted means and 95% confidence intervals, respectively, for each diet (d) per Sampling 2 and nutritional status (feeding and postprandial). For detailed statistically significant information, see Table S8 and Figure S8.

In stomach, *ghrl2*, and particularly *mboat4*, had significantly lower expression than *ghrl1*; the overall level of mRNA expression was 12,531 ± 2605 (*n* = 104), 1160 ± 242 (*n* = 104), and 1.6 ± 0.5 (*n* = 104) for *ghrl1*, *ghrl2*, and *mboat4*, respectively. Stomach samples were only collected on day 56, fish fed all diets were analyzed, and there were no significant differences neither in the effect of diet nor the effect of nutritional status in any gene (Figure 3).

#### 3.6.3. Midgut

**Figure 4:**
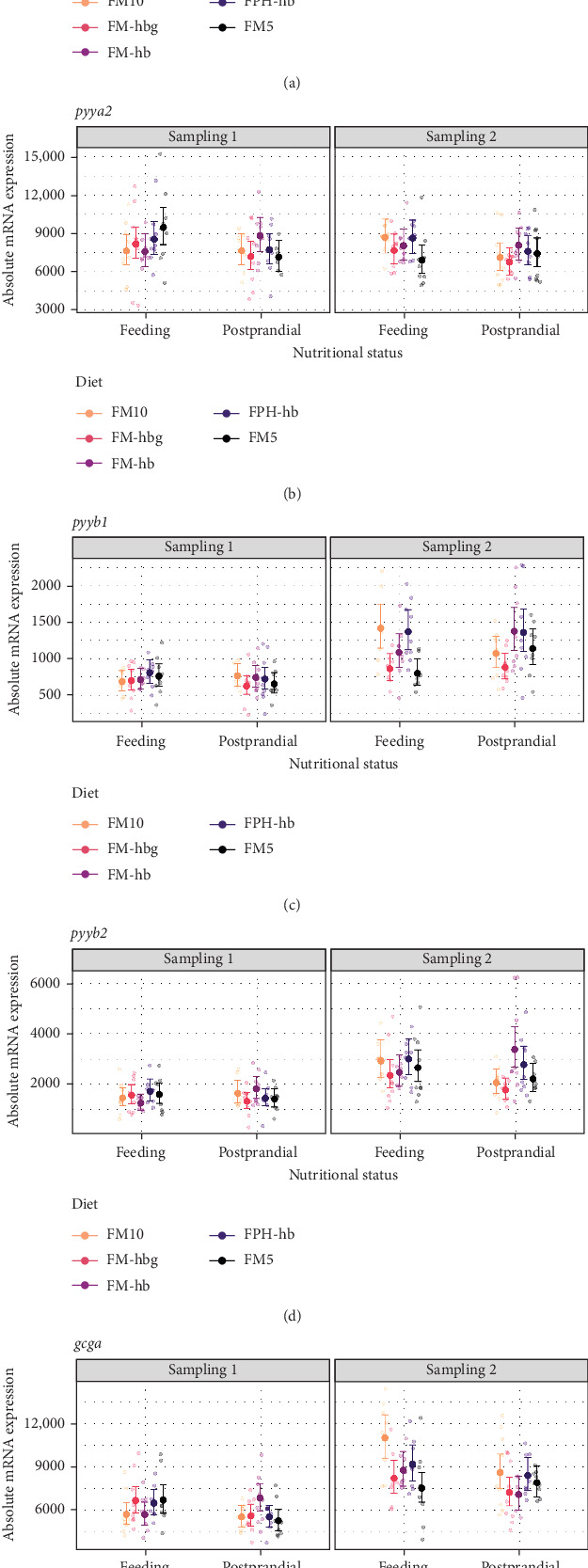
Model prediction of values and raw data of absolute mRNA expression of (a) *peptide YYa*-1 (*pyya1*), (b) *peptide YYa*-2 (*pyya2*), (c) *peptide YYb*-1 (*pyyb1*), (d) *peptide YYb*-2 (*pyyb2*), and (e) *glucagon-a* (*gcga*) in Atlantic salmon midgut (sample size per group = 9). The dots indicate model-predicted means and 95% confidence intervals, respectively, for each diet (d) per sampling (Sampling 1 and Sampling 2) and nutritional status (feeding and postprandial). For detailed statistically significant information, see Table S9 and Figure S9.

Four pyy paralogous genes were identified in the Atlantic salmon genome database in GenBank, and named *pyya1*, *pyya2*, *pyyb1*, and *pyyb2*, in addition to one *gcg* paralog, named *gcga*. The overall level of mRNA expression in the midgut was 4133 ± 1068 (*n* = 175), 7860 ± 1920 (*n* = 176), 918 ± 399 (*n* = 171), 2057 ± 1005 (*n* = 171), and 7219 ± 2156 (*n* = 175) for *pyya1*, *pyya2*, *pyyb1*, *pyyb2*, and *gcga*, respectively. Independent of status or sampling time, there were some similarities in the expression patterns of the five genes analyzed, however, at different expression levels. In feeding fish, there was a significantly (*p* < 0.05) higher expression at day 21 than day 56 of *pyya2* for FM5, and significantly higher expression at day 56 than day 21 of *pyya1* for FM10; of *pyyb1* for FM10, FM-hb, and FPH-hb; of *pyyb2* for all diets analyzed; and for *gcga* for all diets analyzed except FM5 (Figure 4). The pattern was similar for postprandial fish; higher expression at day 56 than day 21 of *pyya1* for FM10; of *pyyb1* for all diets analyzed; of *pyyb2* for FM-hb, FPH-hb, and FM5; and of *gcga* for all diets analyzed except FM-hb. There was an effect of nutritional status at day 21; feeding fish had higher expression than postprandial fish of *pyya2* and *gcga* for FM5, and postprandial fish had higher expression than feeding fish of *pyyb2* for FM-hb. At day 56, feeding fish had higher expression than postprandial fish of *pyyb2* and *gcga* for FM10, and postprandial fish had higher expression than feeding fish of *pyyb1* for FM5. Dietary effect was observed at day 56 feeding fish; FM10 had significantly higher expression of *pyyb1* than FM-hbg, FPH-hb, and FM5; and FPH-hb had significantly higher expression than FM-hbg and FM5 for *pyyb1*. FM10 was significantly higher than FM-hbg and FM5 for *gcga*. While in midgut postprandial fish *pyyb1* was significantly higher in FM-hb and FPH-hb compared to FM-hbg, and *pyyb2* was significantly higher in FM-hb compared to FM10 and FM-hbg.

#### 3.6.4. Hindgut

**Figure 5:**
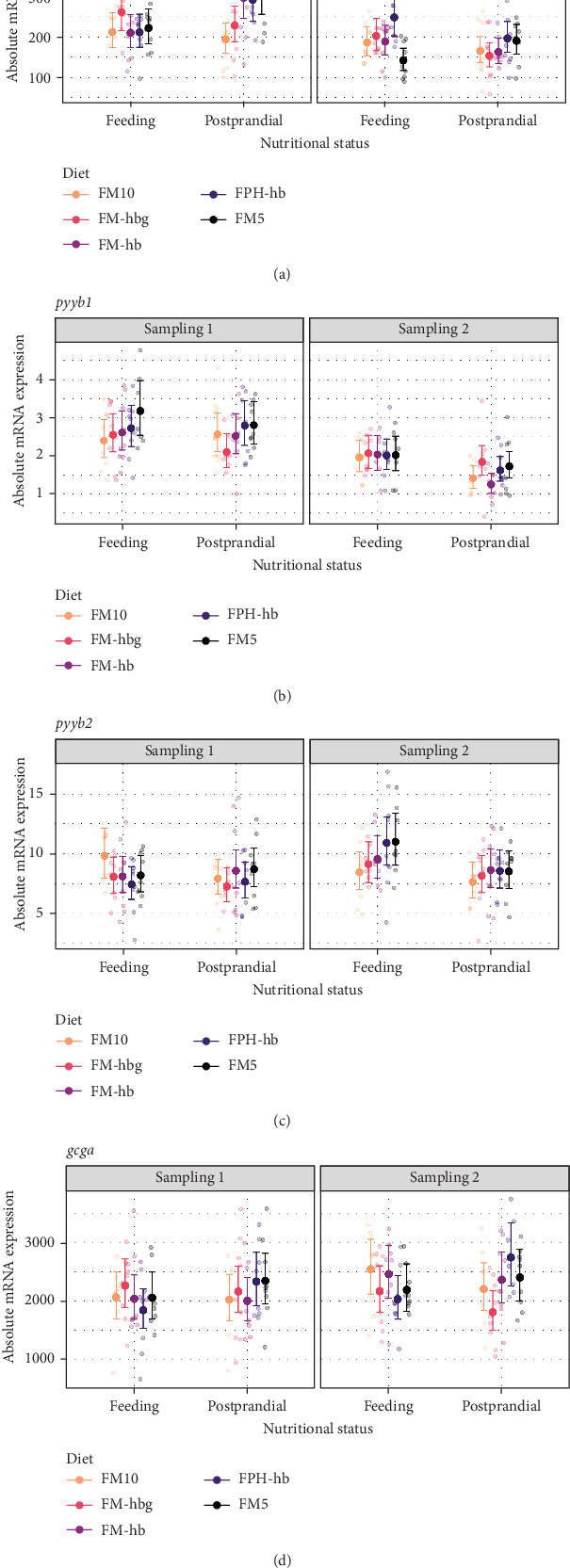
Model prediction of values and raw data of absolute mRNA expression of (a) *peptide YYa*-2 (*pyya2*), (b) *peptide YYb*-1 (*pyyb1*), (c) *peptide YYb*-2 (*pyyb2*), and (d) *glucagon-a* (*gcga*) in Atlantic salmon hindgut (sample size per group = 9). The dots indicate model-predicted means and 95% confidence intervals, respectively, for each diet (d) per sampling (Sampling 1 and Sampling 2) and nutritional status (feeding and postprandial). For detailed statistically significant information, see Table S10 and Figure S10.

The expression levels of *pyy* and *gcg* paralogous genes were also investigated in the hindgut, except for *pyya1* due to its very low expression level in this tissue. Also, the other genes were much lower expressed in hindgut; the overall level of mRNA expression in the hindgut was 213 ± 77 (*n* = 177), 2.2 ± 0.8 (*n* = 165), 8.6 ± 2.5 (*n* = 174), and 2201 ± 629 (*n* = 175) for *pyya2*, *pyyb1*, *pyyb2*, and *gcga* respectively. The mRNA expression level of paralog *pyya1* was at a trace/non-detectable level in the hindgut when compared to the other paralogs, *pyya2*, *pyyb1*, and *pyyb2*. In feeding fish, there was a significantly (*p* < 0.05) higher expression at day 21 than day 56 of *pyya2* for FM5 and *pyyb1* for FPH-hb and FM5, and significantly higher expression at day 56 than day 21 of *pyyb2* for FPH-hb and FM5 (Figure 5). In postprandial fish, there was a significantly (*p* < 0.05) higher expression at day 21 than day 56 of *pyya2* for all analyzed diets except FM10, and for *pyyb1* for all diets except FM-hbg. There was an effect of nutritional status at day 21; postprandial fish had higher expression than feeding fish of *pyya2* for FM-hb, FPH-hb, and FM5. At day 56, feeding fish had higher expression than postprandial fish of *pyya2* for FM-hbg, and of *pyyb1* for FM10 and FM-hb. Postprandial fish had higher expression than feeding fish of *pyya2* for FM5 and *gcga* for FPH-hb. Dietary effect was observed at day 21 postprandial fish; FM10 had significantly lower expression of *pyya2* than the rest of the diets except FM-hbg. At day 56, feeding fish had significantly higher expression of *pyya2* of FPH-hb compared to FM5, and postprandial fish had significantly higher expression of *gcga* for FPH-hb compared to FM-hbg.

## 4. Discussion

In utilizing fish side-stream biomass, besides the numerous raw material specie options, there are also several technological alternatives, as for example the production of traditional FMs, silages, or enzymatic hydrolysates. However, the relative performance of farmed salmon fed such ingredients in the diet is less known. In the present study Atlantic salmon were fed isoenergetic and isonitrogenous diets balanced for EAAs, fatty acids, minerals, vitamins, and other micronutrients. The ingredients supplied different nutrients with possibly different functional effects, where viscera (guts) contributed with a higher content of fat (mainly neutral lipids and high triacylglycerol), low molecular peptides, FAA, and less ash, while heads and backbone contributed with more phospholipids, ash, and larger peptides. The diet content of WSP was only slightly higher with the ingredients with hydrolyzed protein (silage and hydrolysate) due to the inclusion of crystalline AAs, and a higher content of nonessential FAA was observed in feeds with silage. All test ingredients ensured similar FI and performance as the high-quality FM without significant differences, thus, performance results are only discussed in relative terms. The FCRs obtained were relatively low and better or comparable to other growth studies in salmon [11, 13, 22], and this shows that our diets were formulated to balance the requirement of salmon of this size. WSP, low molecular size peptides, and FAA have been shown to affect appetite and growth in farmed fish [11, 12, 22]. In our study, salmon fed the cod viscera silage-containing feed (FPC-g) with the highest level of smaller molecules (<1000 Da), as well as high FAA and water-soluble content showed relatively high FI. For FPC-g the increased FI did not result in higher growth but a relatively higher FCR, and this has also previously been shown in salmon fed diets added putative appetite-stimulating small peptides and FAAs [13]. On the other hand, the FM10 diet had a low content of these assumed appetite-stimulating components but gave a similar high FI as the FPC-g diet, whereas the FM5 diet with the highest level of FAA had the lowest FI, which contradicts the above explanation, or as well suggests other counteracting factors. The relatively high FI in FM10 resulted in higher growth, possibly due to relatively higher ADC of protein, fat, energy, and valine in this diet. Diet FM5 had higher FAA due to addition of crystalline AA, mainly higher lysine, and showed low FCR and high values for PER, as well as higher ADC of several AA compared to FPC-g. Feed with FM containing the cod heads and backbone (FM-hb) gave intermediate FI, but relatively higher growth and lower FCR, in line with results using a similar ingredient with higher MW peptides >10.000 Da [13]. While feed with protein hydrolysate based on cod head and backbone (FPH-hb) gave intermediate FI, slightly lower growth and higher FCR, giving poorer results than a similar ingredient with medium sized peptides [13]. Feed with FM containing the cod head, backbone, and viscera (FM-hbg) gave lower FI, but also lower FCR resulting in only slightly lower growth compared to FM10 and FM-hb. It appears that there was no extra benefit for overall performance exchanging higher MW water-soluble compounds with intermediate and smaller peptides and FAA in this study, different to what was shown in other studies with salmon [13]. Some studies have shown that high FAA can give reduced utilization of dietary AA for anabolic purposes due to deviation in absorption peaks for different crystalline AAs [58, 59]. Other studies suggest that as long as AA profile is similar the protein utilization of the diets should be the same [60]. In the same study they also showed that crystalline AAs and protein-bound AAs are utilized similarly efficiently at crystalline levels <10% [60]. In the current study, the AA profiles were similar for most diets, and also for five of the most known feeding stimulants in fish; glycine, alanine, proline, arginine, and histidine [18], and all diets had moderate levels of crystalline AAs, below 5%. Fish fed FPC-g treatment, however, showed significantly lower ADC of glutamic acid, methionine, isoleucine, and valine compared to diet FM5 in addition to lower ADC of valine compared FM10, and this may have contributed to the poorer performance in the FPC-g diet. Glutamic acid is involved in appetite regulation [61], and higher FI in FPC-g may be associated with lower ADC. Methionine is involved in transport of lipid and energy storage [61], and a relatively higher VSI in FPC-g may be associated with low ADC of methionine. In addition, fish fed FM5 showed significantly higher ADC of lysine compared to the rest of the diets, and the latter may be due to higher crystalline lysine addition in this diet. Even though the novel raw materials had different attributes in the present study, the feed formulation aimed to standardize dietary macro- and micronutrients to known requirement, thus, masking any other differences in feeds than those given by the test ingredients. In previous studies partial and full replacement of marine protein has shown both negative effects, and positive effects on fish FI and performance [15, 62–64]. The minor differences in FCR in this trial may mean that processing of the novel side-stream raw materials preserved the quality of these raw materials improving the protein and fat utilization potential and contributing to the relatively lower FCR in the FM-hb and FM-hbg products. It may also be that the lower FI in some of these diets made the fish utilize the nutrients better, hence, lowering the FCR. Despite few significant differences in the present trial, both protein efficiency ratio (PER) and lipid efficiency ratio (LER) had a highly negative correlation to FCR (*R*^2^ = 0.94, *p*  < 0.0001). Feed efficiency (FE) is the inverse of FCR, that is the biomass realized per unit of feed input. PER and LER are measures of how well the macronutrients support growth and maintenance, and higher values indicate better protein or lipid quality, and is expected to increase FE. There was a strong positive correlation between PER and LER (*R*^2^ = 0.92, *p*  < 0.0001), indicating a well-balanced diet. High protein efficiency is beneficial for farmed fish which require substantial protein for growth, muscle development, and overall health, and high-quality protein from marine origin, including side-stream products, shows positive effects on the metabolic health of both fish and consumers of fish [65]. Nutrient digestibility, retention values, and metabolic rate can be affected by a complex mix of several factors like nutrient level in feed, feeding intensity and level, and pellet quality, as well as whole body status, all part of the momentary picture [66, 67], and the significant difference in nutrient ADC in our study may have been affected by a combination of these factors. A high ash FM may increase the dietary level of several minerals above adequate minimum level [68], thus, decreasing the digestibility of P, like it did for the FM-hb diet in our study. However, mineral and mono-calcium-phosphate (MCP) premixes in formulations made sure all the diets were within the recommended levels, and the levels of dietary P was higher than the estimated dietary P requirement [68]. Also, the observed dietary levels were within the same range as previous studies [69, 70]. There was no correlation between feed P levels or feed soluble P levels and ADC_P_ levels. The feeding regime was the same for all diets, while whole-body composition (WBC) retention of nutrients was not measured in this trial. The yttrium content (Y) of the FPH-hb diet was tenfold range lower than the other diets due to a dosing error, so head and bone hydrolysis effect on ADC was not possible to compare (FM-hb vs. FPH-hb). However, previous studies showed that the P from hydrolyzed bone meal is more available for bone development and growth [71]. In line with performance data there were no significant differences in CF, D%, somatic indexes (SIs), or tissue fat between the diets, and there were no significant differences in ADC of macronutrients or energy between the diets. Reduced disintegration stability in feed can increase palatability and stomach emptying rate, and thus, FI [28, 72–74], making it possible to process more feed through the stomach and gut within a limited amount of time [75, 76]. However, in this trial the feed with the highest stability had the highest FI (FM10) and the lowest stability had the lowest FI (FM5), so water stability seemed to play a lesser role in this trial. Also, the feeds had relatively similar water stability values, except FM5. High pellet hardness, however, has been shown to negatively affect both FI and growth [77] and could be part of the explanation for lower FI and growth for FM5. The trial showed that high growth rates and low FCR can be achieved in Atlantic salmon fed low FM diet (50–100 g kg−1 diet) including side-stream products resulting in low FIFO values (0.44–0.63, corrected for FCR values). Despite few significant differences, there were numerical differences in performance, SIs, and body composition that would give very relevant economic differences for the industry; same growth, 10% lower FCR as well as lower visceral fat and fillet fat in diets with FM-based on head and backbone (FM-hb), in addition to 10% lower FCR and lower visceral fat in diets with FM based on head, backbone, and viscera (FM-hb), both compared to FM control (FM10).

The fish in this trial were conditioned to a frequent feeding regime at constant light, that is, 2–3 min eight times each day with 20% overfeeding. Even though the regime was frequent, the fish were only fed in total 20 min a day with 2 h and 15–30 min between each feeding from 11:00 am to 03:00 am. Feeding in meals instead of continuous reduces the risk of hierarchy in the tanks, as dominant fish will not be able to eat it all. To try to uncover the reason for the few differences observed in terms of FI and performance between the different diets evaluated in this study, we analyzed the mRNA expression levels of key genes involved in appetite control in the whole brain, stomach, midgut, and hindgut. Thus, we investigated how the expression of selected key genes involved in the gut–brain axis [35] were affected by the six different diets, as well as the nutritional status (feeding vs. postprandial), and feeding conditioning over time (day 21 and day 56). Feeding fish were sampled during feeding, meaning fish were expected to still be hungry, while postprandial fish were sampled 1–2 h after the last meal, meaning fish were expected to still be sated after the last meal, and before the next meal the fish were conditioned to expect. The regulatory mechanisms underlying food intake in salmon have been a focus of research in recent years to improve food consumption and nutrient utilization efficiency [31–33]. In the whole brain, the specific paralog *agrp1* was analyzed, while for *npy* and *cart*, the analysis used did not differentiate between different paralogs. In general, *cart* and *npy* genes had a higher expression than *agrp1*. The center of appetite control in vertebrates is the HYP [33], and *agrp1* is only expressed in this region of the brain in the Atlantic salmon [35], while the other genes are expressed in other areas of the brain as well. In feeding fish, there was a significantly (*p* < 0.05) higher mRNA expression of *agrp1* for FM10 on day 21 than on day 56. The expression of orexigenic gene *agrp1* is a sign of hunger and is linked to stimulation of appetite and FI [40, 78, 79], and upregulation of *agrp1* corresponds to cumulative FI data that showed that FM10 from an early stage was the diet giving highest effect on FI. After 8 weeks of feeding the fish should be conditioned to the feeds, and this may be the reason for downregulation on day 56. In the group of feeding fish, a dietary effect was observed at day 21, where fish fed FM10 and FM5 had a significantly higher expression of *agrp1* than FM-hb. The cumulative FI showed that FM10 was highest from start to end of trial, FM5 was lowest from start to finish, while FM-hb FI was in the middle. So regulation of other functions than FI may also affect the level of *agrp1*; the significantly lower *agrp1* in FM-hb might be indirectly related to the significantly lower ADC of P in FM-hb compared to FM10 and FM5, as P is an important metabolic component involved in fundamental pathways including energy metabolism, bone mineralization, and growth [80–82]. During sampling day 21, there was also an effect of nutritional status; feeding fish had significantly higher *agrp1* mRNA expression compared to the postprandial fish for FM10 and FM-hbg, which is as expected, since feeding fish are still not satiated, while after feeding normally orexigenic factors decrease their levels of expression [33, 47]. It may also be that the high frequency feeding regime in this trial in general caused lower expression of brain appetite stimulation neuropeptides. In our study, the postprandial fish were sampled only 1–2 h after feeding, so the appetite-stimulating orexigenic drive was probably not a necessity, while the effect of long-term fasting on hypothalamic mRNA has shown an increase of *agrp1* in several studies including both Atlantic salmon and sea bass [79, 83, 84]. In mammals, *NPY* is highly orexigenic, and the NPY/AGRP-related peptide neurons in the arcuate nucleus (ARC) are vital in the regulation of feeding and energy homeostasis [85, 86]. The expression of orexigenic peptides Npy are linked to stimulation of appetite and FI in salmon [40, 79], while the expression of anorexigenic peptides Cart is linked to satiety and a decrease in appetite and food intake [33, 42, 79]. Some paralogs of neuropeptides Npy and Cart are expressed in the HYP, but also several other brain sections, especially related to feeding and energy status [35], and since we analyzed the mRNA expression in the whole brain, our findings do not account for brain region-specific changes in expression as the regulation of the genes in one compartment could be confounded with regulation in other compartments of the brain [79]. Also, we did not discriminate the *npy* or *cart* paralogs as they were not available at the time of this study. Due to these facts, the expression of *agrp1* may provide the best indication of an effect on appetite control, as this gene paralog is only expressed in the HYP, while total *npy* and *cart* are not discussed in detail. Relevant brain-specific paralogs like *agrp2*, *npya1*, *npya2*, *npyb*, *cart1a*, *1b1*, *1b2*, *2a*, *2b1*, *2b2*, *3a1*, *3a2*, *3b*, and *4*, nor *pomca1*, *pomca2*, and *pomcb* were not included in this study [36–41].

The stomach's function is to store, mix, and break down feed and produce hormones, as well as start the digestion by protein hydrolysis and secure a steady supply of nutrients to the intestine. Ghrl is an important hormone, with functionalities to monitor and control activity locally and to communicate with the brain, and Ghrl, mainly produced and released in the stomach during hunger state, has orexigenic functions both in mammals and several fish species [87–89]. Two paralogs named *ghrl1* and *ghrl2*, in addition to *mboat4*, are identified in Atlantic salmon [44, 52, 53, 90, 91], and in the stomach all these specific paralogs were analyzed in the current study. In the stomach, *ghrl1* had a higher absolute gene expression than *ghrl2* and a much higher expression than *mboat4*. Previous studies showed a low relative expression for *ghrl1* and *ghrl2* in the stomach in the continuous feeding group and slightly higher in 6 days starved group of Atlantic salmon [46], or a high relative expression for *ghrl1* and *ghrl2* in fish fed until afternoon the day before sampling and for fish fasted 4 weeks [53]. In this latter study, *ghrl2* was higher expressed than *ghrl1* and especially *mboat4*. Ghrl is involved in gut motility regulation and growth hormone stimulation, as well as FI stimulation and energy homeostasis [44, 90, 91], and Mboat4 modifies Ghrl to activate growth hormones [44, 88, 90–94]. However, in this study, neither *ghrl1* and *ghrl2*, nor *mboat4* were significantly different as an effect of diet or nutritional status. However, the higher expression of *ghrl1* compared to *ghrl2* could indicate that this gene paralog is more important in salmon. GHRL and low glucose activate several ARC NPY neurons [85], and this relation was supported by our study where *npy* in the brain and *ghrl1* and *ghrl2* in the stomach were highly correlated on an average dietary level (*R*^2^ = 0.97–0.99, polynomial) in postprandial fish. This may mean that there were some differences in stimulation effects between the diets even though there were no significant differences in FI levels. In postprandial fish, on an average dietary level, there was also a high level of correlation between *cart* in the brain and *ghrl1* and *ghrl2* in the stomach (*R*^2^ = 0.86–0.95), and to some degree between *cart* and *npy* in brain and *mboat4* in stomach (*R*^2^ = 0.70 and 0.56, respectively). While in feeding fish, on an average dietary level, there were a high level of correlation between *agrp1* in brain and *ghrl1* in stomach (*R*^2^ = 0.81, polynomial), some correlation between *agrp1* in brain and *mboat4* in stomach (*R*^2^ = 0.56), *cart* in brain and *ghrl2* in stomach (*R*^2^ = 0.64), and between *npy* in brain and *ghrl1* in stomach (*R*^2^ = 0.56). Since Mboat4 activates Ghrl a lower *mboat4* expression in this trial may mean that less Ghrl was acylated; *ghrl1* and *ghrl2* may be at their peak and the low *mboat4* level could mean that salmon started to downregulate appetite stimulation and growth hormone release. It could also mean that their activation levels are different, depending on the signaling pathways involved and the needs of the fish. A study in Atlantic salmon smolt showed that *ghrl1*, but not *ghrl2* was affected by short-term starvation; circulating levels of Ghrl and *ghrl1* mRNA were related to energy metabolism, and *ghrl1* and plasma levels were significantly higher in fasted fish 2 days after feeding [95]. In this study, the feeding fish were sampled during a meal, while the postprandial fish were sampled 1–2 h after the last meal. In Rainbow trout, the return of appetite is correlated with the postprandial stomach time-correlated filling status [75]. Ghrl and the missing significant stomach effects might be explained by the high filling rate due to the high-frequency feeding regime, as it can take 6–12 h to reduce the stomach content by 50% and 24–48 h to empty [96]. In mammals where the orexigenic role of GHRL is clearly established, it is produced in the stomach when emptied between meals, but what happens at high feeding frequencies and if the stomach will be continuously filled, is that the hunger signals from the stomach to brain are probably not induced (decreased). Studies in both sheep and humans have shown reduced response of GHRL at increased meal frequencies [97, 98]. The latter study also showed that insulin and ghrl plasma concentrations normally are inversely related, but at high meal frequencies, this relationship was not observed [98].

The main function of the intestine is to digest the feed, absorb nutrients and water, and discard waste. Pyy and Gcg and their gene paralogs in fish may be primary biomarkers for and involved in digestion, but they also signal a downregulation of appetite. Pyy, a hormone mainly secreted from the midgut and hindgut, intestine EEC have an anorexigenic function acting on the HYP in both mammals and fish and is involved in the downregulation of food intake and body weight [99–103], as well as gut motility and secretion [104]. GCG is a precursor for a group of hormones synthesized in the pancreas, distal intestine, and colon in mammals, that is, GLP-1 and GLP-2, and GCG and insulin hormones are involved in glucose homeostasis (feedback system) [105, 106]. In this study, we identified four *pyy* gene paralogs named *pyya1*, *pyya2*, *pyyb1*, and *pyyb2* and one *gcg* paralog named *gcga* in midgut and hindgut, and to our knowledge, the *pyy* and *gcga* paralogs analyzed in midgut and hindgut have not previously been reported for Atlantic salmon [107, 108]. The data suggest a notable variation in the expression levels of the paralogs, with *gcga* and *pyya2* being more dominant in the midgut. In the hindgut, *gcga* exhibited the highest mRNA expression levels, significantly outpacing other paralogous genes. These findings highlight *gcga* as the most highly expressed gene in the hindgut, with *pyyb1* showing the lowest detectable expression. The paralog *pyya1* was expressed at a trace/non-detectable level in the hindgut. Opposite of what we see in mammals [101, 109], and as observed previously in Atlantic salmon [46], all *pyy* paralogs were much more abundant and *gcga* higher in the midgut than the hindgut in this study. In mammals, *pyy* is mainly expressed in the hindgut and is known to inhibit food intake [99–103]. The reason for the generally higher levels of gene expression in midgut could be that satiety signal early in the gut is more important in salmon or that fish has a more rapid feedback mechanism compared to mammals. Also, it could be a result of the momentary local function of sensing and adjusting the adequate response according to what type of components arriving from stomach and pyloric cecal compartments. Previous studies showed a low relative expression for *pyy* in midgut of continuous feeding group and even lower in 6 days starved fish group of Atlantic salmon [46]. In the postprandial fish, the *pyy* paralog levels were expected to be higher, which was not a clear pattern in this trial neither per diet and per gene paralog nor in general, as their levels were in the same ranges with few significant differences both in midgut and hindgut. Also, there were no clear dietary differences or pattern, maybe except a dietary effect observed during sampling on day 21 in hindgut postprandial fish with lower *pyya2* expression in FM10 compared to FM-hb, FPH-hb, and FM5. For fish fed FM10 and FM-hb the opposite pattern was observed as compared to the brain and may be an anorexigenic response to the previous upregulation of *agrp1* in the brain for feeding fish fed FM10, and downregulation of *agrp1* in the brain for feeding fish fed FM-hb. Comparing sampling points, the midgut expression of most *pyy* paralogs were significantly higher on day 56 than day 21, while in hindgut some *pyy* paralogs were higher on day 21 compared to day 56, and this shift may mean that the potentially more active gene paralogs in midgut are conditioned over time to respond faster than in hindgut. In mammals, PYY seems to inhibit gastrointestinal motility and pancreatic exocrine activity, and it may be similar for salmon and other teleosts [33, 110]; in this trial, the lack of differential expression might indicate that the anorexic signals already have been relayed and *pyy* downregulated again in the postprandial fish. Glucagon having an anorexigenic role is involved in glucose homeostasis by stimulating the conversion of glycogen stored in the liver to glucose to be released into the bloodstream [106], and recent studies show that it also modulates lipid metabolism [110]. In humans, both GLP-1 and GLP-2 enhance satiety and reduce food intake. GLP-1 stimulates insulin secretion from the pancreas and inhibits glucagon secretion, lowering blood glucose levels, and GLP-2 stimulates growth and repair of the intestinal lining and increases the absorption of nutrients from the intestines [106, 111, 112]. Therefore, we would have expected the precursor *gcga* levels to be higher in the postprandial fish, which was not a clear pattern in this trial either per diet or in general; the expression of *gcga* was in the same ranges in feeding and postprandial fish with few significant differences both in midgut and hindgut. It may be that the anorexic signals already were sent and *gcg* production was reduced because of the finalization of the meal. It may also be that the active role of Glp-1 and Glp-2 in the enhancement of satiety and reduction of food intake is not the same in salmon as in humans [106, 111, 112]. The upregulation of several NPY neurons from GHRL and low glucose is counteracted by leptin and insulin downregulating the same neurons [85]. In this trial, we did not analyze blood plasma, so it was not possible to find the glucose status for feeding and postprandial fish, or correlations with mRNA expression in the brain or gut segments. The *gcga* expression was lower in hindgut compared to the midgut, as with the expression *pyy* paralogs. Comparing samplings, the expression of *gcga* was significantly higher on day 56 than day 21 in midgut in both feeding and postprandial fish for most diets, and this may be due to conditioning over time. A dietary effect was seen at day 56 in the group of feeding fish midgut with significantly higher *gcga* expression for FM10 than FM-hbg and FM5, and this may be related to the significantly lower expression of *cart* in FM10 compared to FM5. Gene expression patterns may be affected by different feeding regimes, time span since last meal, dietary composition, and individual fish differences, and since most trials only have one to two sampling points the expression levels may be a result of complex interactions between factors [46].

Merging all diets together, the gene expression level in feeding fish was slightly higher than in postprandial fish, opposite of what would be expected. The reason for this could be that there are delays from gene expression and new synthesis of protein expression and neuropeptide nerve signals regulating the specific functionality, making it difficult to map the momentary picture [47, 113], meaning that high levels during feeding was because the fish started to be satiated. We analyzed the gene expression level of several signaling substances in different parts of the gut that we assume have key roles in regulating digestion in salmon. We did not manage to get a clear picture of how these factors affect the complex and dynamic interactions that take place between different GIT regions as part of the digestive processes. Although, we found correlations between gene paralogs in stomach and whole brain neuropeptide expression. There was no clear relationship between the intestine hormone mRNA expression and the whole brain neuropeptide expression, which likely is a consequence of using the whole brain in the analyses instead of only the HYP. The data gives a certain insight into the regulation of the digestive process in Atlantic salmon; however, the differences between the diets were probably too small to have large effects on the modulation of the signaling factors and management of the digestive process. The analyzed values, however, represent reference values for status in salmon with good appetite and high growth. The main function of the brain and digestive system is to utilize and maximize the macronutrients and micronutrients received as it is moved through the GIT, and given the similar end results for fish fed the different diets the gut–brain axis performed its job despite differences in raw material composition in the diets.

In the present experiment Atlantic salmon were fed diets including different ingredients produced from cod side-streams processed into FM, protein hydrolysate, or silage, using combinations of heads, backbones, and viscera (guts), substituting 50% of the high-quality protein contribution of a conventional FM or equal part of high-quality plant-based ingredients. All the feeds showed similar FI and comparable growth, and the lack of significant differences in FCR as well as lipid, protein, and energy digestibility between dietary groups suggests that incorporating side-stream materials has minimal impact on digestive regulation and performance compared to FM-based diets. These results showed that more sustainable FM and hydrolysates (FM-hbg, FM-hb, FPH-hb, and FPC-g) based on side-stream products can partly replace FM in diets for Atlantic salmon potentially giving more efficient and sustainable feeds for salmon for optimal performance. It is important to choose raw materials with high-quality proteins for optimized nutrient efficiency, and overall high performance, and the use of novel raw materials based on side-stream products will increase sustainability in aquaculture. Also, a more environmentally sustainable feed production needs to focus on effective feeding strategies and low FCR as well as growth, to reduce the loss of nitrogen waste to sea [114]. In addition to reduced eutrophication, a lower FCR means less feed for the same growth, and it will also boost revenue in aquaculture.

## 5. Conclusions

Overall, this study demonstrates that raw materials based on side-stream products from the Atlantic cod industry with varying characteristics can partially replace high-quality commercial FMs and vegetable proteins, delivering comparable performance while enhancing the sustainability of the feeds. Data indicated that *agrp1* may be a biomarker for appetite and that *pyya1*, *pyya2*, and *gcga* in midgut may be possible biomarkers for the digestive process and for postprandial satiety in Atlantic salmon.

## Figures and Tables

**Table 1:** Diet composition of marine ingredients (marine protein DM basis, %).

Composition of experimental ds	FM10	FM-hbg	FM-hb	FPH-hb	FPC-g	FM5
Ingredient marine protein (%DM)	—	3.42	3.42	3.42	3.42	—
Marine protein total (%DM)	6.83	6.83	6.83	6.83	6.83	3.42

Abbreviations: d, diet; DM, dry matter; FM, fish meal; FPC, fish protein concentrate; FPH, fish protein hydrolysate.

**Table 2:** Raw material composition for the diets (%, as is).

Composition of experimental ds	FM10	FM-hbg	FM-hb	FPH-hb	FPC-g	FM5
FM SA superprime	10.00	5.00	5.00	5.00	5.00	5.00
FM-hbg	—	5.54	—	—	—	—
FM-hb	—	—	5.39	—	—	—
FPH-hb	—	—	—	4.86	—	—
FPC-g	—	—	—	—	13.74	—
Soy protein concentrate	9.00	9.00	8.50	8.50	9.00	9.00
Sunflower expeller	6.84	6.11	2.00	2.00	6.27	3.86
Wheat gluten	9.00	9.30	10.60	10.80	9.00	10.80
Maize gluten	10.00	10.00	10.00	10.00	10.00	10.00
Pea protein	10.00	10.00	10.00	10.00	10.00	10.00
Guar meal	10.00	10.00	10.00	10.00	10.00	10.00
Wheat	11.09	11.10	11.00	11.05	11.00	11.05
Fish oil	13.57	13.38	13.85	13.77	12.42	13.85
Rapeseed oil	5.81	5.74	5.90	5.90	5.32	5.94
AA mix^*∗*^	1.96	1.88	2.40	2.30	2.14	4.55
Vit and min premix	0.44	0.44	0.44	0.44	0.44	0.44
MCP	2.40	2.60	2.48	2.66	2.34	2.83
Choline chloride	0.28	0.26	2.55	2.55	0.26	2.55
Water change^*∗∗*^	−0.52	−0.47	−0.24	0.08	−7.05	−0.08

Abbreviations: AA, amino acid; d, diet; DM, dry matter; FM, fish meal; FPC, fish protein concentrate; FPH, fish protein hydrolysate; MCP, mono-calcium-phosphate.

*⁣* ^*∗*^Histidine, threonine, methionine, and lysine.

*⁣* ^*∗∗*^The term “Water change” is used in formulations to make sure raw materials are included/excluded in the ds based on nutritional value and not based on moisture content. Moisture will be both added and removed during the processing of the ds, and the final product will add up to 100% based on the defined feed DM in formulations.

**Table 3:** Sequence of specific primers used for reverse transcriptase quantitative PCR (RT-qPCR) analysis.

Gene	Gene Bank Acc. number	Primer sequences 5′−3′	Efficiency (%)	*R* ^2^	References
agrp1	NM_001146677.1 XM_014182676.1 XM_014182677.1	F: GCGTTCTCCCCGTCGCTGTA R: TGTTAGGGGCGCCTGTGAGC	97.6	0.999	Murashita et al. [36]
cart	NM_001146680.1 XM_014183838.2	F: AGCAACTGCTTGGAGCACTACATGAC R: CAGTCGCACATTTTGCCGATTCTCGCGCCC	98.5	0.995	Murashita et al. [36]
npy	NM_001146681.1 XM_014178359.1	F: ACTGGCCAAGTATTACTCCGCTCTCA R: CTGTGGGAGCGTGTCTGTGCTCTCCTTC	96.7	1.000	Murashita et al. [36]
pyya1	XM_014204561.1	F: GTCTTCTCAAAATGGCCGTTATGC R: AGTCGTCATATCTTGATCTCTGGT	96.6	0.991	Designed by Authors
pyya2	NM_001139523.1	F: GTCTTCTCAAAATGGCCATGATAC R: AGTCGTCATATCTTGATCTCTGGT	95.2/92.4	0.999/0.998	Designed by Authors
pyyb1	XM_014157007.1 XM_014157008.1	F: CCGCCCAAGCCTGTGAGCCC R: CGTCTCCAAACAGCAACCACG	108.9/97.7	0.996/1.000	Designed by Authors
pyyb2	XM_014159227.1	F: GACAGAGGTATGGAAAGAGGTCTG R: TGGGACGGGACCCAGATTTA	103.6/99.9	0.995/0.993	Designed by Authors
gcga	XM_014150353.2 XM_014151823.2	F: GAATGGTCCAATGAGCAGAAG R: TCAATGATCTTCTTGACGTTT	96.6/101.5	0.998/0.997	Designed by Authors
ghrl1	NM_001142709.1	F: CCAGAAACCACAGGTAAGACAGGGTA R: GAGCCTTGATTGTATTGTGTTTGTCT	90.6	0.996	Del Vecchio et al. [52]
ghrl2	NM_001139585.1	F: TCCCAGAAACCACAGGGTAAA R: GAGCCTTGATTGTATTGTGTTTGTCT	95.6	0.987	Del Vecchio et al. [52]
mboat4	XM_045703012.1	F: GGGTTGGCAAA—CATTCTGGC R: ACACTGATAGGAGAAGCCTGG	97.5	1.000	Kalananthan et al. [53]

*Note:* Sequence accession number (Ensemble and GenBank databases), primer sequences, qPCR efficiency (%), and *R*^2^ are indicated for each primer pair.

Abbreviations: Acc., accession; agrp1, agouti-related protein-1; cart, cocaine- and amphetamine-regulated transcript; gcga, glucagon-a; ghrl, ghrelin; mboat4, membrane-bound O-acyltransferase domain-containing 4; npy, neuropeptide Y; pyya, peptide YYa; pyyb, peptide YYb.

**Table 4:** Chemical composition of ingredients produced from cod side-stream products (g/100 g sample).

Chemical composition ingredients	FM-hbg	FM-hb	FPH-hb	FPC-g
Water (%)	7.3	7.9	1.8	51.1
Crude protein (*N* × 6.25; %)	61.6	63.3	70.2	25.0
Crude fat (%)	13.3	3.5	4.3	15.7
Ash (%)	16.2	26.7	25.2	5.9
P (%)	3.94	4.12	4.11	0.74
Soluble P (%)	0.71	0.65	0.56	0.23
WSP (%)	30.8	20.7	49.7	23.2
Peptide size in WSP (% of sample)				
MW > 20,000 Da	3.63	8.76	0.45	0.07
MW 20,000–15,000 Da	1.39	2.26	0.20	0.12
MW 15,000–10,000 Da	2.28	2.44	0.35	0.35
MW 10,000–8000 Da	1.29	0.87	0.50	0.23
MW 8000–6000 Da	1.60	0.83	1.64	0.30
MW 6000–4000 Da	1.85	0.56	4.42	0.39
MW 4000–2000 Da	1.91	0.37	9.64	0.51
MW 2000–1000 Da	1.02	0.14	9.19	0.67
MW 1000–500 Da	0.92	0.10	7.90	1.48
MW 500–200 Da	1.48	0.14	6.51	4.22
MW < 200 Da	13.43	4.24	8.95	14.85
Sum AAs (%)	53.0	56.4	62.1	21.5
Sum EAAs (%)	23.1	24.0	26.2	9.7
Sum FAAs (%)	9.8	1.6	4.5	8.4

*Note:* See Tables S1 and S2 for extensive analysis of the ingredient composition.

Abbreviations: AAs, amino acids; EAAs, essential amino acids; FAAs, free amino acids; FM, fish meal; FPC, fish protein concentrate; FPH, fish protein hydrolysate; MW, molecular weight; P, phosphorous; WSP, water-soluble protein.

**Table 5:** Chemical composition of the experimental diets (g/100 g sample).

Chemical composition ds	FM10	FM-hbg	FM-hb	FPH-hb	FPC-g	FM5
DM (%)	95.8	96.9	98.1	95.9	97.0	96.3
Crude protein (*N* × 6.25; %)	45.6	45.5	47.8	46.5	46.8	46.2
Crude fat (%)	24.8	24.5	25.2	24.3	24.1	24.4
Gross energy (kJ/g)	23.3	23.5	24.0	23.2	23.5	23.5
P (%)	1.25	1.34	1.33	1.36	1.24	1.25
Soluble P (%)	0.93	1.02	0.88	0.93	0.98	1.03
WSP (%)	7.5	7.8	8.1	9.8	9.8	9.6
Peptide size in WSP (% of sample)						
MW > 20,000 Da	0.23	0.27	0.41	0.25	0.22	0.21
MW 20,000–15,000 Da	0.24	0.25	0.30	0.27	0.25	0.27
MW 15,000–10,000 Da	0.68	0.69	0.75	0.86	0.81	0.88
MW 10,000–8000 Da	0.38	0.38	0.43	0.47	0.44	0.52
MW 8000–6000 Da	0.41	0.41	0.43	0.52	0.48	0.54
MW 6000–4000 Da	0.40	0.40	0.39	0.59	0.46	0.50
MW 4000–2000 Da	0.41	0.41	0.40	0.77	0.45	0.48
MW 2000–1000 Da	0.30	0.30	0.30	0.67	0.35	0.37
MW 1000–500 Da	0.38	0.39	0.41	0.74	0.50	0.51
MW 500–200 Da	0.41	0.50	0.53	0.74	0.88	0.68
MW < 200 Da	3.71	3.81	3.75	3.90	4.94	4.65
Sum AA (%)	41.9	42.3	44.0	41.3	42.5	40.9
Sum EAA (%)	19.1	19.1	19.9	18.5	19.5	19.4
Sum FAA total (%)	2.34	2.60	2.13	2.63	3.22	3.98
Sum FAA not added (%)	0.44	0.78	0.53	0.57	1.25	0.37
Sum FAA added^*∗*^ (%)	1.90	1.82	1.60	2.06	1.97	3.61
WSP–FAA added (%)	5.60	5.98	6.50	7.74	7.83	5.99

*Note:* See Tables S3 and S4 for extensive analysis of the d composition.

Abbreviations: AA, amino acid; d, diet; DM, dry matter; EAA, essential amino acid; FAA, free amino acid; FM, fish meal; FPC, fish protein concentrate; FPH, fish protein hydrolysate; MW, molecular weight; P, phosphorous; WSP, water-soluble protein.

*⁣* ^*∗*^Histidine, threonine, methionine, and lysine (added).

**Table 6:** Physical pellet quality (PPQ) of the diets (sample basis).

Physical composition ds	FM10	FM-hbg	FM-hb	FPH-hb	FPC-g	FM5	LM (*p* <)
Pellet hardness (N)	38.6^b,c^	34.9^b^	38.8^b,c^	34.8^b^	30.3^a^	43.8^c^	0.0001
WSI (%)	75.2^c^	72.6^b,c^	71.9^b,c^	73.5^b,c^	68.0^b^	60.5^a^	0.0001
Total porosity (%)	38.1^a,b^	33.0^a^	36.2^a^	43.6^b^	38.0^a,b^	38.3^a,b^	0.0067

*Note:* Mean ± stdev; *n* = 20 (Kahl), *n* = 3 (WSI, porosity). Statistical analysis by linear modeling (LM) and Tukey's post hoc test, *p*  < 0.05. Different letters indicate significant differences between the diets. See Figures S1 and S2 for Micro-CT pore structure and distribution analysis.

Abbreviations: d, diet; FM, fish meal; FPC, fish protein concentrate; FPH, fish protein hydrolysate; ns, nonsignificant; WSI, water stability index.

**Table 7:** Fish growth and feeding performance data.

Fish performance per d	FM10	FM-hbg	FM-hb	FPH-hb	FPC-g	FM5	LM (*p* <)
Start fish #*r*	100	100	100	100	100	100	ns
Mortality (%)	0.7 ± 1.2	0	0	1.3 ± 1.2	2 ± 2.6	1.7 ± 2.1	ns
Initial weight (g), day 0	113 ± 1	113 ± 1	112 ± 0	112 ± 1	113 ± 0	113 ± 1	ns
Mid weight (g), day 21	174 ± 20	179 ± 46	167 ± 7	170 ± 7	166 ± 26	164 ± 6	ns
Final weight (g), day 56	275 ± 11	270 ± 13	274 ± 12	266 ± 10	266 ± 5	264 ± 11	ns
Total FI (kg)	9.9 ± 0.7	8.8 ± 0.7	9.1 ± 0.4	8.9 ± 1.8	9.3 ± 0.6	8.4 ± 0.4	ns
FI (% mbw/day)^1^	1.30 ± 0.16	1.18 ± 0.18	1.20 ± 0.02	1.19 ± 0.19	1.26 ± 0.06	1.16 ± 0.02	ns
FCR^2^	0.73 ± 0.05	0.66 ± 0.05	0.66 ± 0.02	0.68 ± 0.11	0.72 ± 0.01	0.67 ± 0.03	ns
SGR^3^	1.60 ± 0.06	1.57 ± 0.08	1.61 ± 0.06	1.56 ± 0.03	1.54 ± 0.04	1.53 ± 0.06	ns
TGC (×10^3^)^4^	2.38 ± 0.11	2.33 ± 0.14	2.40 ± 0.10	2.32 ± 0.07	2.28 ± 0.06	2.27 ± 0.10	ns
PER^5^	2.98 ± 0.21	3.28 ± 0.24	3.10 ± 0.11	3.17 ± 0.53	2.91 ± 0.06	3.19 ± 0.16	ns
LER^5^	5.55 ± 0.38	6.05 ± 0.44	5.99 ± 0.21	6.12 ± 1.02	5.74 ± 0.12	6.12 ± 0.30	ns
FIFO^6^ (FCR = 1)	0.86	0.67	0.69	0.68	0.63	0.69	—
FIFO^6^ (FCR = FCR^2^)	0.63	0.44	0.45	0.46	0.46	0.46	—

*Note:* Mean ± stdev; *n* = 3. Statistical analysis by linear modeling (LM) and Tukey's post hoc test, *p*  < 0.05.

Abbreviations: d, diet; FCR, feed conversion ratio; FI, feed intake; FIFO, fish-in fish-out ratio; FM, fish meal; FPC, fish protein concentrate; FPH, fish protein hydrolysate; LER, lipid efficiency ratio; ns, nonsignificant; PER, protein efficiency ratio; SGR, specific growth rate; TGC, thermal growth coefficient.

^1^FI is the mean feed consumption per fish per day as a % of the daily fish body weight.

^2^FCR is feed consumed/biomass increase.

^3^SGR is (ln BW_2_−ln BW_1_)/feeding days × 100.

^4^TGC is (BW^1/3^_2_−BW^1/3^_1_) × 1000/Ʃ (temp. (°C) × feeding days), according to Cho [57].

^5^PER and LER is fish weight gain/nutrient consumption.

^6^FIFO is (level of FM in the d (%) + level of fish oil in the d (%))/(yield of FM from wild fish + yield of fish oil from wild fish) × FCR.

**Table 8:** ADC of stripped content from hindgut.

Apparent digestibility per d	FM10	FM-hbg	FM-hb	FPH-hb	FPC-g	FM5	LM (*p* <)
Total lipid (%)^1^	86.3 ± 1.2	84.9 ± 0.3	84.8 ± 2.7	—	83.0 ± 1.9	86.3 ± 0.5	ns
Crude protein (%)^1^	86.8 ± 0.9	85.6 ± 0.5	84.8 ± 2.2	—	84.6 ± 0.7	87.1 ± 0.9	ns
Energy (kJ/g)^1^	75.6 ± 1.5	72.1 ± 1.2	71.0 ± 3.9	—	71.7 ± 1.2	73.3 ± 0.8	ns
Total P (%)^1^	48.8 ± 2.6^b^	44.9 ± 2.0^a,b^	37.4 ± 2.3^a^	—	42.8 ± 3.2^a,b^	49.7 ± 6.2^b^	0.0116

*Note:* Mean ± stdev; *n* = 3. Statistical analysis by linear modeling (LM) and Tukey's post hoc test, *p*  < 0.05. Different letters indicate significant differences between the diets. See Table S5 for extensive ADC analysis for AAs.

Abbreviations: ADC, apparent digestibility coefficient; d, diet; f, feaces; FM, fish meal; FPC, fish protein concentrate; FPH, fish protein hydrolysate; *N*, nutrient content; ns, nonsignificant; P, phosphorous; Y, yttrium content.

^1^ADC is 100−10 × *Y*_d_ × *N*_f_/*N*_d_/*Y*_f_.

**Table 9:** Fish biometrics and tissue composition on day 56 (in g kg^−1^ unless otherwise specified).

Fish biometrics per d	FM10	FM-hbg	FM-hb	FPH-hb	FPC-g	FM5	LM (*p* <)
CF^1^	1.23 ± 0.08	1.20 ± 0.07	1.21 ± 0.04	1.22 ± 0.03	1.21 ± 0.05	1.18 ± 0.04	ns
D%^2^	88.1 ± 1.1	89.4 ± 0.3	89.0 ± 0.9	88.9 ± 0.8	88.6 ± 1.3	88.7 ± 0.7	ns
VSI^3^	10.51 ± 0.99	9.43 ± 0.34	9.74 ± 0.77	9.83 ± 0.84	10.16 ± 1.22	10.02 ± 0.65	ns
HSI^3^	1.22 ± 0.11	1.08 ± 0.03	1.11 ± 0.13	1.10 ± 0.09	1.15 ± 0.12	1.16 ± 0.05	ns
CSI^3^	0.15 ± 0.02	0.13 ± 0.02	0.15 ± 0.02	0.14 ± 0.02	0.13 ± 0.02	0.14 ± 0.02	ns
GB%^4^	0.10 ± 0.04	0.10 ± 0.04	0.10 ± 0.03	0.09 ± 0.02	0.09 ± 0.04	0.10 ± 0.04	ns
Muscle fat (%)	22.5 ± 2.4	23.5 ± 4.5	20.8 ± 3.3	23.6 ± 1.9	23.6 ± 0.9	25.1 ± 3.3	ns
Visceral fat (%)	5.0 ± 0.2	4.8 ± 0.2	4.9 ± 0.6	5.4 ± 1.0	4.9 ± 0.2	4.7 ± 0.4	ns
Liver fat (%)	9.5 ± 0.4	10.3 ± 0.2	10.0 ± 0.5	9.9 ± 0.6	9.8 ± 0.1	9.5 ± 0.4	ns

*Note:* Mean ± stdev; *n* = 6. Statistical analysis by linear modeling (LM) and Tukey's post hoc test, *p*  < 0.05. See Table S6 for biometrics Day 21. VSI: viscera excluding liver and heart.

Abbreviations: CF, condition factor; CSI, cardio somatic index; d, diet; D%, dress-out percentage; FM, fish meal; FPC, fish protein concentrate; FPH, fish protein hydrolysate; GB%, gallbladder percentage; HSI, hepatosomatic index; ns, nonsignificant; SI, somatic index; VSI, viscera somatic index.

^1^CF is fish weight (g) × fish fork length^−3^ (cm) × 100.

^2^D% is gutted fish weight/whole fish weight × 100.

^3^SI are tissue weight/whole fish weight × 100.

^4^GB% is gallbladder weight/whole fish weight × 100.

## Data Availability

The data for this article are available upon reasonable request to the corresponding author.

## Supporting Information

Supporting Information Extensive analysis of the ingredient composition of AAs, FAA, nucleotides, FA, and lipid classes is presented in Tables S1 and S2. Extensive analysis of the experimental feed composition of AAs, FAA, FA, and lipid classes is presented in Tables S3 and S4. Micro-CT pore structure and distribution analysis of the experimental feeds are presented in Figures S1 and S2. Atlantic salmon ADC values for AAs are presented in Tables S5. Atlantic salmon biometrics from Day 21 is presented in Tables S6. Extensive statistical analysis of gene expression from whole brain, stomach, midgut, and hindgut are presented in Tables S7–S10, respectively, and overall expression patterns in Figures S3–S6, respectively. Random effect of tank in gene expression models are presented in Figures S7–S10, respectively, for whole brain, stomach, midgut, and hindgut.
