# Supplementary material for: Side-Stream Based Marine Solubles From Atlantic Cod (Gadus morhua) Modulate Appetite and Dietary Nutrient Utilization in Atlantic Salmon (Salmo salar L.) and can Replace Fish Meal
Source: Aquac Nutr. 2025 Jan 30;2025:4872889. doi: 10.1155/anu/4872889 (PMC11824393; doi:10.1155/anu/4872889)

# Supplementary Material

**TABLE S1** Protein analysis of total amino acids (AA) and free amino acids (FAA) (g/100g protein), and nucleotides (mg/kg) in ingredients from cod side-stream-products.

| **AA (g/100g protein)** | **FM-hbg** | **FM-hb** | **FPH-hb** | **FPC-g** |  | **FAA (g/100g protein)** | **FM-hbg** | **FM-hb** | **FPH-hb** | **FPC-g** |
| --- | --- | --- | --- | --- | --- | --- | --- | --- | --- | --- |
| **Aspartic**  **acid** | 7.8 | 8.1 | 8.0 | 7.7 |  | ***Creatinine*** | 0.31 | 0.35 | 0.63 | 0.88 |
| **Glutamic**  **acid** | 11.9 | 12.2 | 12.1 | 11.9 |  | ***Aspartic***  ***acid*** | 0.76 | 0.05 | 0.16 | 1.84 |
| **Serine** | 4.7 | 4.9 | 4.8 | 4.5 |  | ***Glutamic acid*** | 1.23 | 0.17 | 0.26 | 2.48 |
| **Glycine** | 9.6 | 10.9 | 11.1 | 8.7 |  | ***Serine*** | 0.76 | 0.06 | 0.20 | 1.72 |
| **Histidine** | 1.6 | 1.7 | 1.7 | 2.0 |  | ***Asparagine*** | 0.02 | 0.00 | 0.03 | 0.76 |
| **Arginine** | 5.5 | 6.6 | 6.6 | 5.8 |  | ***Glycine*** | 0.73 | 0.09 | 0.19 | 2.12 |
| **Threonine** | 4.1 | 3.6 | 3.6 | 4.0 |  | ***Glutamine*** | 0.37 | 0.02 | 0.11 | 0.40 |
| **Alanine** | 6.0 | 6.2 | 6.3 | 5.7 |  | ***3-Amino-***  ***1-propanol*** | 0.06 | 0.13 | 0.14 | 0.08 |
| **Proline** | 5.2 | 5.8 | 5.7 | 5.0 |  | ***Taurine*** | 2.11 | 0.92 | 0.87 | 1.92 |
| **Tyrosine** | 3.4 | 3.2 | 3.1 | 3.8 |  | ***Histidine*** | 0.13 | 0.00 | 0.06 | 0.44 |
| **Valine** | 4.4 | 3.9 | 3.8 | 4.4 |  | ***4-Amino butyric acid*** | 0.29 | 0.02 | 0.01 | 0.04 |
| **Methionine** | 2.9 | 3.0 | 3.0 | 2.9 |  | ***Citrulline*** | 0.08 | 0.02 | 0.01 | 0.12 |
| **Isoleucine** | 3.6 | 3.3 | 3.3 | 3.6 |  | ***Threonine*** | 0.80 | 0.05 | 0.17 | 1.72 |
| **Leucine** | 6.5 | 6.0 | 5.8 | 6.4 |  | ***Alanine*** | 1.22 | 0.14 | 0.41 | 2.28 |
| **Phenylalanine** | 3.4 | 3.3 | 3.1 | 3.5 |  | ***Carnosine*** | 0.00 | 0.00 | 0.00 | 0.16 |
| **Lysine** | 5.5 | 6.3 | 6.4 | 6.2 |  | ***Arginine*** | 0.37 | 0.03 | 0.30 | 2.40 |
|  |  |  |  |  |  | ***Proline*** | 0.71 | 0.05 | 0.14 | 0.76 |
| **EAA** | **37.5** | **37.9** | **37.3** | **38.7** |  | ***Anserine*** | 0.06 | 0.02 | 0.07 | 0.12 |
|  |  |  |  |  |  | ***Tyrosine*** | 0.70 | 0.03 | 0.17 | 1.40 |
| **Nucleotides (mg/kg)** |  |  |  |  |  | ***Valine*** | 0.97 | 0.06 | 0.28 | 1.28 |
| **Hypoxanthine** | 2300 | 1400 | 1100 | 1300 |  | ***Methionine*** | 0.55 | 0.05 | 0.17 | 1.60 |
| **Inosine Monophosphate (IMP)** | 550 | 37 | 290 | 25 |  | ***Isoleucine*** | 0.76 | 0.03 | 0.34 | 1.00 |
| **Inosin** | 2300 | 1500 | 1600 | 400 |  | ***Leucine*** | 1.56 | 0.08 | 1.01 | 3.52 |
| **Adenosine Monophosphate (AMP)** | 280 | 160 | 340 | 35 |  | ***Phenylalanine*** | 0.75 | 0.03 | 0.41 | 1.84 |
| **Adenosine Diphosphate (ADP)** | 67 | 50 | 110 | 74 |  | ***Tryptophan*** | 0.18 | 0.00 | 0.04 | 0.12 |
| **Adenosine Triphosphate (ATP)** |  |  |  |  |  | ***Lysine*** | 0.36 | 0.06 | 0.26 | 2.64 |
|  |  |  |  |  |  |  |  |  |  |  |
| **Nucleotide K-value** | **91.0** | **95.9** | **86.8** | **97.0** |  | ***FAA*** | **15.93** | **2.45** | **6.47** | **33.68** |

**TABLE S2** Fatty acids (FA) and lipid classes (g/100g extracted crude fat) in ingredients from cod side-stream-products.

| **FA (g/100 fat)** | **FM-hbg** | **FM-hb** | **FPH-hb** | **FPC-g** |  | **Lipid classes (g/100g fat)** | **FM-hbg** | **FM-hb** | **FPH-hb** | **FPC-g** |
| --- | --- | --- | --- | --- | --- | --- | --- | --- | --- | --- |
| **14:0** | 3.5 | 1.2 | 0.9 | 3.6 |  | ***Triacylglycerol*** | 50.0 | 11.0 | 7.5 | 88.0 |
| **16:0** | 10.4 | 10.6 | 9.5 | 12.3 |  | ***Diacylglycerol*** | 3.1 | 0.6 |  | 2.2 |
| **18:0** | 2.3 | 2.4 | 2.3 | 2.9 |  | ***Free fatty acids*** | 14.0 | 13.0 | 11.0 | 2.7 |
| **Sum saturated fatty acids** | 16.2 | 14.3 | 12.7 | 18.8 |  | ***Cholesterol*** | 3.2 | 13.0 | 12.0 | 0.7 |
| **16:1 n-7** | 5.4 | 1.6 | 1.2 | 5.6 |  | ***Cholesterol esters*** | 3.6 | 11.0 | 13.0 | 0.6 |
| **18:1 (n-9)+(n-7)+(n-5)** | 16.4 | 11.3 | 10 | 20 |  | ***Phosphatidylethanolamin*** | 1.6 | 6.5 | 6.4 |  |
| **20:1 (n-9)+(n-7)** | 10.6 | 4.1 | 3.5 | 10.6 |  | ***Phosphatidilcholin*** | 3.1 | 25.0 | 30.0 |  |
| **22:1 (n-11)+(n-9)+(n-7)** | 5.5 | 1.9 | 1.5 | 6.1 |  | ***Lyso-phosphatidylcholin*** |  |  | 0.6 |  |
| **24:1 n-9** | 0.5 | 0.8 | 0.7 | 0.5 |  | ***Total polar lipids*** | 4.6 | 31.9 | 36.6 | 0.9 |
| **Sum monoenoic fatty acids** | 38.4 | 19.7 | 16.9 | 42.8 |  | ***Totale neutral lipids*** | 74.2 | 49.0 | 44.1 | 94.3 |
| **18:2 n-6** | 1.4 | 0.7 | 0.6 | 1.3 |  | ***Total lipids*** | 78.8 | 81.0 | 80.7 | 95.2 |
| **20:2 n-6** | 0.2 | 0.2 | 0.1 | 0.2 |  |  |  |  |  |  |
| **20:3 n-6** | 0.1 | 0.1 | 0.1 | 0.1 |  |  |  |  |  |  |
| **20:4 n-6** | 0.6 | 1.3 | 1.1 | 0.4 |  |  |  |  |  |  |
| **Sum PUFA (n-6) fatty acids** | 2.3 | 2.4 | 1.9 | 2.0 |  |  |  |  |  |  |
| **18:3 n-3** | 0.5 | 0.2 | 0.1 | 0.8 |  |  |  |  |  |  |
| **18:4 n-3** | 1.5 | 0.5 | 0.3 | 2.1 |  |  |  |  |  |  |
| **20:4 n-3** | 0.4 | 0.3 | 0.2 | 0.5 |  |  |  |  |  |  |
| **20:5 n-3 EPA** | 6.2 | 6.7 | 5.4 | 7.9 |  |  |  |  |  |  |
| **21:5 n-3** | 0.2 | 0.1 | 0.1 | 0.2 |  |  |  |  |  |  |
| **22:5 n-3** | 0.7 | 1.8 | 1.5 | 0.7 |  |  |  |  |  |  |
| **22:6 n-3 DHA** | 11.0 | 15.6 | 12.8 | 10.6 |  |  |  |  |  |  |
| **Sum PUFA (n-3) fatty acids** | 20.6 | 25.2 | 20.4 | 22.9 |  |  |  |  |  |  |
| **Sum total-PUFA fatty acids** | 23.2 | 27.8 | 22.4 | 25.2 |  |  |  |  |  |  |
| **Sum identified fatty acids** | 77.8 | 61.8 | 52.0 | 86.8 |  |  |  |  |  |  |

**TABLE S3** Protein analysis of total (AA) and free amino acid (FAA) composition of experimental feeds (g/100g feed) with ingredients from cod side-stream-products.

| **AA (g/100g feed)** | **FM10** | **FM- hbg** | **FM-hb** | **FPH-hb** | **FPC-g** | **FM5** |  | **FAA (g/100g feed)** | **FM10** | **FM- hbg** | **FM-hb** | **FPH-hb** | **FPC-g** | **FM5** |
| --- | --- | --- | --- | --- | --- | --- | --- | --- | --- | --- | --- | --- | --- | --- |
| **Aspartic acid** | 3.6 | 3.6 | 3.7 | 3.4 | 3.6 | 3.2 |  | ***Creatinine*** | 0.07 | 0.07 | 0.11 | 0.08 | 0.11 | 0.06 |
| **Glutamic acid** | 9 | 9.3 | 9.7 | 9.2 | 9.2 | 9 |  | **Aspartic acid** | 0.02 | 0.04 | 0.02 | 0.02 | 0.08 | 0.01 |
| **Hydroksyproline** | 0 | 0 | 0 | 0 | 0 | 0 |  | **Glutamic acid** | 0.03 | 0.05 | 0.03 | 0.03 | 0.09 | 0.02 |
| **Serine** | 2.1 | 2.1 | 2.2 | 2.1 | 2.1 | 1.9 |  | ***Serine*** | 0.01 | 0.03 | 0.01 | 0.02 | 0.06 | 0.01 |
| **Glycine** | 1.9 | 2 | 2.1 | 2 | 2 | 1.6 |  | ***Asparagine*** | 0.02 | 0.02 | 0.02 | 0.02 | 0.04 | 0.02 |
| **Histidine** | 1.3 | 1.3 | 1.4 | 1.3 | 1.4 | 1.3 |  | ***Glycine*** | 0.01 | 0.03 | 0.01 | 0.02 | 0.08 | 0.01 |
| **Arginine** | 2.8 | 2.8 | 2.9 | 2.7 | 2.8 | 2.5 |  | ***Glutamine*** | 0.01 | 0.01 | 0.01 | 0.01 | 0.01 | 0.01 |
| **Threonine** | 1.6 | 1.6 | 1.7 | 1.6 | 1.7 | 1.6 |  | ***Taurine*** | **0.06** | **0.10** | **0.08** | **0.07** | **0.11** | **0.04** |
| **Alanine** | 2.1 | 2 | 2 | 1.9 | 2 | 1.8 |  | ***4-Amino butyric acid*** | 0.01 | 0.02 | 0.01 | 0.01 | 0.01 | 0.01 |
| **Proline** | 2.8 | 2.9 | 3 | 2.9 | 2.8 | 2.8 |  | ***Alanine*** | **0.04** | **0.07** | **0.04** | **0.05** | **0.11** | **0.03** |
| **Tyrosine** | 1.3 | 1.3 | 1.4 | 1.3 | 1.3 | 1.2 |  | ***Arginine*** | **0.04** | **0.06** | **0.05** | **0.05** | **0.12** | **0.04** |
| **Valine** | 1.9 | 1.9 | 2 | 1.8 | 1.9 | 1.8 |  | ***Proline*** | **0.01** | **0.03** | **0.01** | **0.01** | **0.03** | **0.01** |
| **Methionine** | 1.1 | 1.1 | 1.1 | 1 | 1.1 | 1 |  | ***Tyrosine*** | 0.01 | 0.03 | 0.01 | 0.02 | 0.04 | 0.01 |
| **Isoleucine** | 1.8 | 1.8 | 1.8 | 1.7 | 1.8 | 1.7 |  | ***Valine*** | 0.01 | 0.04 | 0.02 | 0.02 | 0.06 | 0.01 |
| **Leucine** | 3.7 | 3.7 | 3.8 | 3.5 | 3.7 | 3.5 |  | ***Isoleucine*** | 0.01 | 0.03 | 0.01 | 0.02 | 0.04 | 0.01 |
| **Phenylalanine** | 2.1 | 2.1 | 2.2 | 2.1 | 2.1 | 2 |  | ***Leucine*** | **0.03** | **0.07** | **0.03** | **0.06** | **0.14** | **0.03** |
| **Lysine** | 2.8 | 2.8 | 3 | 2.8 | 3 | 4 |  | ***Phenylalanine*** | **0.02** | **0.04** | **0.02** | **0.03** | **0.07** | **0.02** |
| **sum AA** | **41.9** | **42.3** | **44** | **41.3** | **42.5** | **40.9** |  | ***Tryptophan*** | 0.01 | 0.01 | 0.01 | 0.01 | 0.01 | 0.01 |
| **sum EAA** | **19.1** | **19.1** | **19.9** | **18.5** | **19.5** | **19.4** |  | ***Ornithine*** | 0.00 | 0.01 | 0.01 | 0.01 | 0.01 | 0.01 |
| **EAA/NEAA** | **0.84** | **0.82** | **0.83** | **0.81** | **0.85** | **0.90** |  | ***sum FAA*** | **0.44** | **0.78** | **0.53** | **0.57** | **1.25** | **0.37** |
|  |  |  |  |  |  |  |  | ***Histidine*** | 0.35 | 0.34 | 0.28 | 0.38 | 0.35 | 0.40 |
|  |  |  |  |  |  |  |  | ***Threonine*** | 0.23 | 0.21 | 0.17 | 0.26 | 0.25 | 0.32 |
|  |  |  |  |  |  |  |  | ***Methionine*** | 0.40 | 0.44 | 0.36 | 0.43 | 0.43 | 0.49 |
|  |  |  |  |  |  |  |  | ***Lysine*** | 0.92 | 0.83 | 0.79 | 0.99 | 0.94 | 2.40 |

**TABLE S4** Fatty acids (FA) and lipid classes composition of experimental feeds (g/100g feed) with ingredients from cod side-stream-products ingredients.

| **Fatty acids (g/100 feed)** | **FM10** | **FM- hbg** | **FM-hb** | **FPH-hb** | **FPC-g** | **FM5** | **Lipid classes (g/100g feed)** | **FM10** | **FM-hbg** | **FM-hb** | **FPH-hb** | **FPC-g** | **FM5** |
| --- | --- | --- | --- | --- | --- | --- | --- | --- | --- | --- | --- | --- | --- |
| **14:0** | 1.01 | 1.01 | 1.10 | 1.00 | 0.95 | 1.01 | ***Triacylglycerol*** | 24.54 | 24.29 | 24.58 | 23.91 | 23.33 | 23.86 |
| **16:0** | 3.39 | 3.47 | 3.51 | 3.51 | 3.52 | 3.37 | ***Free fatty acids*** | 0.30 | 0.46 | 0.54 | 0.24 | 0.46 | 0.32 |
| **18:0** | 0.73 | 0.73 | 0.77 | 0.73 | 0.75 | 0.74 | ***Cholesterol esters*** | 0.15 | 0.13 | 0.15 | 0.12 | 0.00 | 0.12 |
| **Sum saturated fatty acids** | 5.26 | 5.36 | 5.53 | 5.39 | 5.35 | 5.26 | ***Phosphatidylethanolamin*** | 0.13 | 0.18 | 0.00 | 0.00 | 0.00 | 0.15 |
| **16:1 n-7** | 1.01 | 1.04 | 1.05 | 1.05 | 1.04 | 1.01 | ***Total polar lipids*** | 0.20 | 0.28 | 0.10 | 0.02 | 0.12 | 0.15 |
| **18:1 (n-9)+(n-7)+(n-5)** | 6.00 | 6.22 | 6.32 | 6.34 | 6.34 | 6.03 | ***Totale neutral lipids*** | 25.10 | 25.02 | 25.50 | 24.38 | 24.18 | 24.48 |
| **20:1 (n-9)+(n-7)** | 0.40 | 0.46 | 0.44 | 0.41 | 0.56 | 0.42 |  |  |  |  |  |  |  |
| **22:1 (n-11)+(n-9)+(n-7)** | 0.38 | 0.35 | 0.41 | 0.34 | 0.41 | 0.37 |  |  |  |  |  |  |  |
| **24:1 n-9** | 0.08 | 0.08 | 0.10 | 0.07 | 0.07 | 0.10 |  |  |  |  |  |  |  |
| **Sum monoenoic fatty acids** | 7.87 | 8.15 | 8.32 | 8.22 | 8.43 | 7.92 |  |  |  |  |  |  |  |
| **18:2n-6** | 2.73 | 2.86 | 3.02 | 2.88 | 2.87 | 2.80 |  |  |  |  |  |  |  |
| **20:2 n-6** | 0.03 | 0.03 | 0.05 | 0.02 | 0.05 | 0.02 |  |  |  |  |  |  |  |
| **20:4n-6** | 0.18 | 0.18 | 0.18 | 0.20 | 0.19 | 0.17 |  |  |  |  |  |  |  |
| **Sum PUFA (n-6) fatty acids** | 3.01 | 3.16 | 3.33 | 3.17 | 3.18 | 3.08 |  |  |  |  |  |  |  |
| **18:3n-3** | 0.78 | 0.78 | 0.87 | 0.81 | 0.73 | 0.79 |  |  |  |  |  |  |  |
| **18:4 n-3** | 0.28 | 0.25 | 0.31 | 0.24 | 0.27 | 0.30 |  |  |  |  |  |  |  |
| **20:4 n-3** | 0.10 | 0.10 | 0.10 | 0.10 | 0.10 | 0.10 |  |  |  |  |  |  |  |
| **20:5n-3 EPA** | 2.02 | 2.02 | 2.20 | 2.05 | 1.94 | 2.04 |  |  |  |  |  |  |  |
| **21:5 n-3** | 0.08 | 0.08 | 0.10 | 0.07 | 0.07 | 0.10 |  |  |  |  |  |  |  |
| **22:5 n-3** | 0.30 | 0.30 | 0.33 | 0.32 | 0.29 | 0.32 |  |  |  |  |  |  |  |
| **22:6n-3 DHA** | 1.57 | 1.47 | 1.72 | 1.44 | 1.41 | 1.53 |  |  |  |  |  |  |  |
| **Sum PUFA (n-3) fatty acids** | 5.14 | 5.03 | 5.66 | 5.03 | 4.84 | 5.17 |  |  |  |  |  |  |  |
| **Sum total-PUFA fatty acid** | 8.45 | 8.48 | 9.32 | 8.49 | 8.26 | 8.54 |  |  |  |  |  |  |  |
| **Sum identified fatty acids** | 22.34 | 21.99 | 23.17 | 22.11 | 22.04 | 21.72 |  |  |  |  |  |  |  |

**FIGURE S1** Micro CT scan of one pellet from feeds FM10, FM-hbg, FM-hb, FPH-hb, FPC-g and FM5. Black spots within the pellet represent air while white, light grey and dark grey represent bone fragments, pellet structure and oil residues respectively. The total porosity (%) was higher for FPH-hb and lower for FM-hbg, and there were differences in pore size and numbers between the diets.


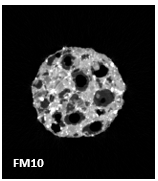

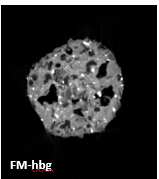

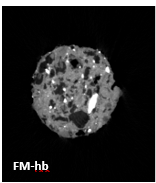

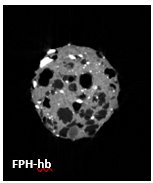

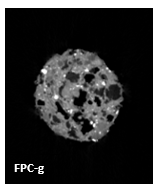

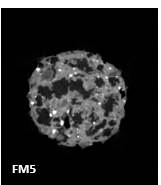


**FIGURE S2** Micro CT scan of feeds FM10, FM-hbg, FM-hb, FPH-hb, FPC-g and FM5 (n=3) showing structure separation distributions (um) vs Pore volume (%). There were differences in pore volume distribution between the diets.*
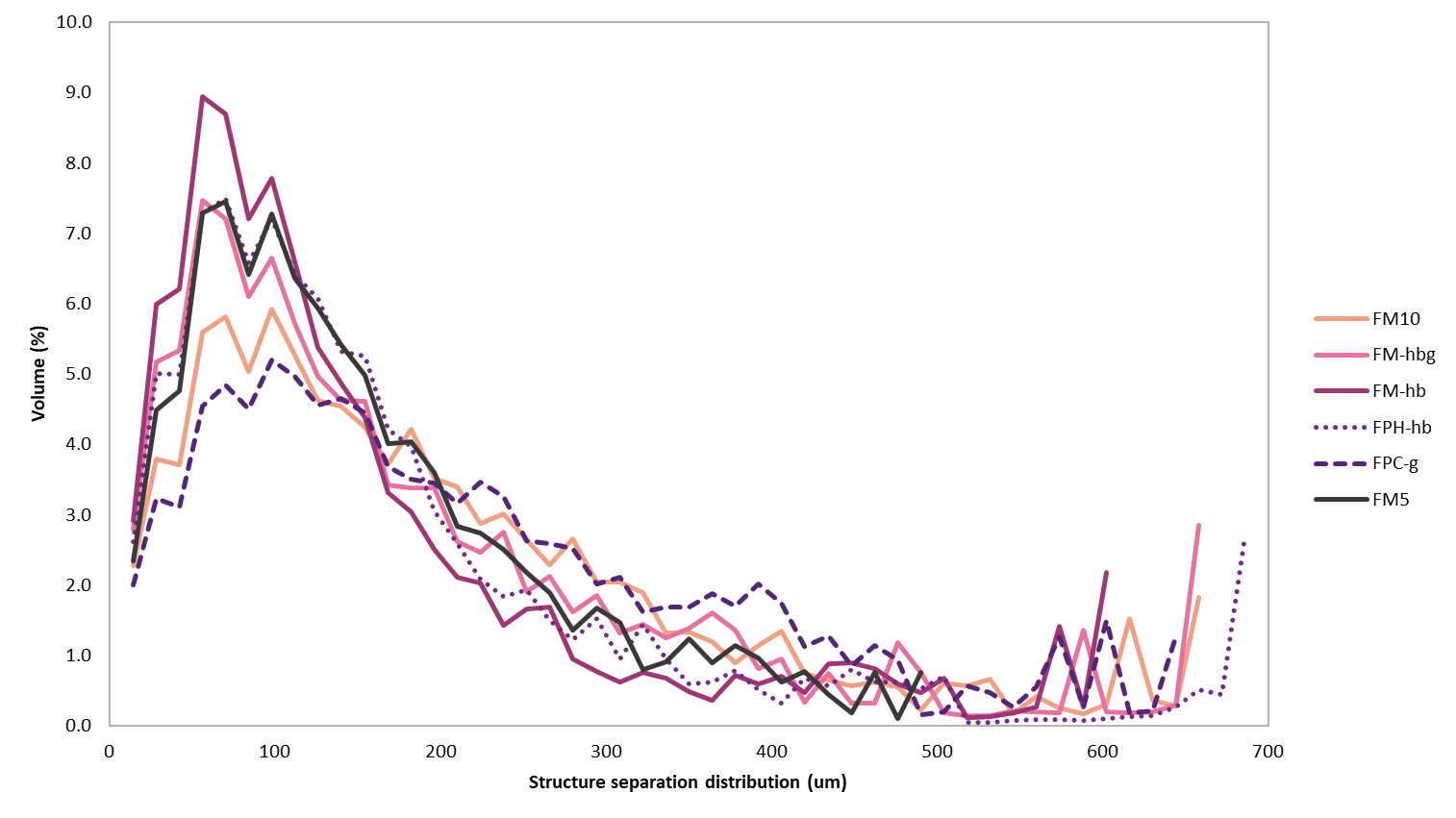
*

**TABLE S5** Apparent digestibility coefficient (ADC) of amino acids (AA) of stripped content from hindgut of Atlantic salmon fed cod side-stream-products. Mean ± stdev; n=3. Statistical analysis by linear modelling (LM) assuming a gaussian distribution as a function of diet, followed by Tukey post hoc pairwise tests. *P*<0.05. ns, non-significant.

| **ADC** |  | **FM10** |  | **FM-hbg** |  | **FM-hb** |  | **FPH-hb** |  | **FPC-g** |  | **FM5** |  | **LM (<P),**  **Diet** |  |
| --- | --- | --- | --- | --- | --- | --- | --- | --- | --- | --- | --- | --- | --- | --- | --- |
| **Aspartic acid** |  | 81.0 |  | 80.1 |  | 78.9 |  |  |  | 76.7 |  | 81.1 |  | ns |  |
| **Glutamic acid** |  | 93.4^ab^ |  | 93.3^ab^ |  | 93.0^ab^ |  |  |  | 91.9^a^ |  | 94.0^b^ |  | 0.0473 |  |
| **Serine** |  | 88.7^ab^ |  | 88.0^ab^ |  | 87.2^ab^ |  |  |  | 86.1^a^ |  | 88.8^b^ |  | 0.0438 |  |
| **Glycine** |  | 83.3 |  | 82.1 |  | 81.4 |  |  |  | 80.9 |  | 83.2 |  | ns |  |
| **Histidine** |  | 91.7 |  | 90.4 |  | 91.3 |  |  |  | 90.1 |  | 92.1 |  | ns |  |
| **Arginine** |  | 95.0 |  | 94.6 |  | 94.4 |  |  |  | 93.6 |  | 94.5 |  | ns |  |
| **Threonine** |  | 86.6 |  | 84.8 |  | 85.3 |  |  |  | 84.0 |  | 85.8 |  | ns |  |
| **Alanine** |  | 88.8 |  | 87.9 |  | 87.0 |  |  |  | 86.1 |  | 88.6 |  | ns |  |
| **Proline** |  | 91.9 |  | 91.4 |  | 90.7 |  |  |  | 89.7 |  | 92.1 |  | ns |  |
| **Tyrosine** |  | 87.8 |  | 87.5 |  | 87.3 |  |  |  | 85.3 |  | 87.6 |  | ns |  |
| **Valine** |  | 88.5^b^ |  | 88.0^ab^ |  | 87.6^ab^ |  |  |  | 85.7^a^ |  | 89.1^b^ |  | 0.0202 |  |
| **Methionine** |  | 92.8^ab^ |  | 92.6^ab^ |  | 92.0^ab^ |  |  |  | 91.4^a^ |  | 93.3^b^ |  | 0.0205 |  |
| **Isoleucine** |  | 90.4^ab^ |  | 90.1^ab^ |  | 89.2^ab^ |  |  |  | 88.1^a^ |  | 90.8^b^ |  | 0.0329 |  |
| **Leucine** |  | 91.6 |  | 91.4 |  | 90.9 |  |  |  | 89.9 |  | 92.0 |  | ns |  |
| **Phenylalanine** |  | 89.8 |  | 89.5 |  | 89.1 |  |  |  | 88.0 |  | 90.1 |  | ns |  |
| **Lysine** |  | 91.5^a^ |  | 90.4^a^ |  | 90.9^a^ |  |  |  | 90.1^a^ |  | 94.0^b^ |  | 0.0018 |  |

**TABLE S6** Day 21 measures of condition factor (CF), dressout percentage (D%), visceral somatic index (VSI), hepatosomatic index (HSI), cardio somatic index (CSI), and from gallbladder percentage (GB%) of body weight Atlantic salmon fed diets with inclusion of cod side-stream-products, feeding and postprandial fish together. Mean ± stdev; n=6. Statistical analysis by linear modelling (LM) assuming a gaussian distribution as a function of diet, followed by Tukey post hoc pairwise tests. *P*<0.05. ns, non-significant.

| **Sampling** | **Status** | **Biometric** | **FM10** | **FM-hbg** | **FM-hb** | **FPH-hb** | **FPC-g** | **FM5** | **LM (<P), Diet** |
| --- | --- | --- | --- | --- | --- | --- | --- | --- | --- |
| **Day 21** | ***Feeding +***  ***Post-prandial*** | ***CF*** | 1.20 ± 0.07 | 1.19 ± 0.05 | 1.21 ± 0.04 | 1.21 ± 0.07 | 1.17 ± 0.04 | 1.15 ± 0.08 | ns |
|  |  | ***D%*** | 88.4 ± 0.8 | 89.2 ± 0.8 | 89.3 ± 1.2 | 88.9 ± 1.2 | 89.1 ± 0.4 | 89.0 ± 1.0 | ns |
|  |  | ***VSI*** | 10.29 ± 0.67 | 9.51 ± 0.76 | 9.47 ± 1.14 | 9.84 ± 1.14 | 9.74 ± 0.44 | 9.61 ± 0.91 | ns |
|  |  | ***HSI*** | 1.18 ± 0.13 | 1.11 ± 0.07 | 1.14 ± 0.10 | 1.15 ± 0.08 | 1.07 ± 0.07 | 1.21 ± 0.15 | ns |
|  |  | ***CSI*** | 0.13 ± 0.02 | 0.14 ± 0.02 | 0.13 ± 0.02 | 0.13 ± 0.01 | 0.13 ± 0.02 | 0.13 ± 0.02 | ns |
|  |  | ***GB%*** | 0.10 ± 0.04 | 0.08 ± 0.04 | 0.08 ± 0.04 | 0.07 ± 0.03 | 0.07 ± 0.03 | 0.08 ± 0.04 | ns |

**TABLE S7** Day 21 and Day 56 whole brain mRNA absolute gene expression of *agrp1*, *cart* and *npy* in Atlantic salmon fed diets with inclusion of cod side-stream-products, feeding and postprandial fish. Mean ± stdev; n=3. Statistical analysis by generalized linear mixed model (GLMM) assuming a gamma distribution as a function of diet, nutritional status and sampling, followed by Tukey post hoc pairwise tests. *P*<0.05. ns, non-significant.

| **Sampling** | **Status** | **Genes** | **FM10** | **FM-hbg** | **FM-hb** | **FPH-hb** | **FPC-g** | **FM5** | **GLMM (<P),**  **Diet** |
| --- | --- | --- | --- | --- | --- | --- | --- | --- | --- |
| **Day 21** | ***Postprandial*** | ***agrp1*** | 0.6 | 0.5 | 0.9 | 0.9 | n.a. | 0.8 | ns |
|  |  | ***cart*** | 143^*^ | 90^*^ | 131 | 107 | n.a. | 137 | ^*^trend |
|  |  | ***npy*** | 126 | 94 | 114 | 100 | n.a. | 137 | ns |
|  | ***Feeding*** | ***agrp1*** | 1.3^b^ | 1.0 | 0.4^a^ | 0.8 | n.a. | 1.5^b^ | 0.014 |
|  |  | ***cart*** | 157^b^ | 133 | 94^a^ | 129 | n.a. | 122 | 0.0304 |
|  |  | ***npy*** | 174^b^ | 145^*^ | 92 ^a,*^ | 139 | n.a. | 124 | 0.0021 / ^*^trend |

| **Sampling** | **Genes** | **Status** | **FM10** | **FM-hbg** | **FM-hb** | **FPH-hb** | **FPC-g** | **FM5** |
| --- | --- | --- | --- | --- | --- | --- | --- | --- |
| **Day 21** | ***agrp1*** | ***Postprandial*** | 0.6^x^ | 0.5^x^ | 0.9^*^ | 0.9 | n.a. | 0.8 |
|  |  | ***Feeding*** | 1.3^y^ | 1.0^y^ | 0.4^*^ | 0.8 | n.a. | 1.5 |
|  | ***cart*** | ***Postprandial*** | 143 | 90^x^ | 131^*^ | 107 | n.a. | 137 |
|  |  | ***Feeding*** | 157 | 133^y^ | 94^*^ | 129 | n.a. | 122 |
|  | ***npy*** | ***Postprandial*** | 126^*^ | 94^x^ | 114 | 100^*^ | n.a. | 137 |
|  |  | ***Feeding*** | 174^*^ | 145^y^ | 92 | 139^*^ | n.a. | 124 |
| **GLMM (<P),**  **Status** | ***agrp1*** |  | 0.0195 | 0.0332 | ^*^trend | ns |  | ns |
|  | ***cart*** |  | ns | 0.0285 | ^*^trend | ns |  | ns |
|  | ***npy*** |  | ^*^trend | 0.0143 | ns | ^*^trend |  | ns |

| **Sampling** | **Status** | **Genes** | **FM10** | **FM-hbg** | **FM-hb** | **FPH-hb** | **FPC-g** | **FM5** | **GLMM (<P),**  **Diet** |
| --- | --- | --- | --- | --- | --- | --- | --- | --- | --- |
| **Day 56** | ***Postprandial*** | ***agrp1*** | 0.5 | 0.7 | 0.9 | 1.1 | n.a. | 0.6 | ns |
|  |  | ***cart*** | 149 | 119 | 122 | 126 | n.a. | 155 | ns |
|  |  | ***npy*** | 105 | 131 | 110 | 124 | n.a. | 145 | ns |
|  | ***Feeding*** | ***agrp1*** | 0.5 | 1.0 | 0.7 | 1.2 | n.a. | 0.9 | ns |
|  |  | ***cart*** | 114^a,*^ | 179^*^ | 134 | 159 | n.a. | 190^b^ | 0.0281 / ^*^trend |
|  |  | ***npy*** | 101 | 132 | 141 | 133 | n.a. | 150 | ns |

| **Sampling** | **Genes** | **Status** | **FM10** | **FM-hbg** | **FM-hb** | **FPH-hb** | **FPC-g** | **FM5** |
| --- | --- | --- | --- | --- | --- | --- | --- | --- |
| **Day 56** | ***agrp1*** | ***Postprandial*** | 0.5 | 0.7 | 0.9 | 1.1 | n.a. | 0.6 |
|  |  | ***Feeding*** | 0.5 | 1.0 | 0.7 | 1.2 | n.a. | 0.9 |
|  | ***cart*** | ***Postprandial*** | 149 | 119^x^ | 122 | 126 | n.a. | 155 |
|  |  | ***Feeding*** | 114 | 179^y^ | 134 | 159 | n.a. | 190 |
|  | ***npy*** | ***Postprandial*** | 105 | 131 | 110 | 124 | n.a. | 145 |
|  |  | ***Feeding*** | 101 | 132 | 141 | 133 | n.a. | 150 |
| **GLMM (<P),**  **Status** | ***agrp1*** |  | ns | ns | ns | ns |  | ns |
|  | ***cart*** |  | ns | 0.020 | ns | ns |  | ns |
|  | ***npy*** |  | ns | ns | ns | ns |  | ns |

**TABLE S8** Day 56 stomach mRNA absolute gene expression of *ghrl1*, *ghrl2* and *mboat4* in Atlantic salmon fed diets with inclusion of cod side-stream-products, feeding and postprandial fish. Mean ± stdev; n=3. Statistical analysis by generalized linear mixed model (GLMM) assuming a gamma distribution as a function of diet, nutritional status and sampling, followed by Tukey post hoc pairwise tests. *P*<0.05. ns, non-significant.

| **Sampling** | **Status** | **Genes** | **FM10** | **FM-hbg** | **FM-hb** | **FPH-hb** | **FPC-g** | **FM5** | **GLMM (<P),**  **Diet** |
| --- | --- | --- | --- | --- | --- | --- | --- | --- | --- |
| **Day 56** | ***Postprandial*** | ***ghrl1*** | 12284 | 11814 | 12030 | 11806 | 12939 | 14645 | ns |
|  |  | ***ghrl2*** | 1140 | 1144 | 1107 | 1095 | 1203 | 1281 | ns |
|  |  | ***mboat4*** | 1.7 | 1.3 | 1.7 | 1.6 | 1.4 | 1.7 | ns |
|  | ***Feeding*** | ***ghrl1*** | 12439 | 12536 | 12598 | 12064 | 12607 | 12789 | ns |
|  |  | ***ghrl2*** | 1160 | 1189 | 1126 | 1079 | 1240 | 1175 | ns |
|  |  | ***mboat4*** | 1.6 | 1.6 | 1.9 | 1.6 | 1.3 | 1.7 | ns |

| **Sampling** | **Genes** | **Status** | **FM10** | **FM-hbg** | **FM-hb** | **FPH-hb** | **FPC-g** | **FM5** |
| --- | --- | --- | --- | --- | --- | --- | --- | --- |
| **Day 56** | ***ghrl1*** | ***Postprandial*** | 12284 | 11814 | 12030 | 11806 | 12939 | 14645 |
|  |  | ***Feeding*** | 12439 | 12536 | 12598 | 12064 | 12607 | 12789 |
|  | ***ghrl2*** | ***Postprandial*** | 1140 | 1144 | 1107 | 1095 | 1203 | 1281 |
|  |  | ***Feeding*** | 1160 | 1189 | 1126 | 1079 | 1240 | 1175 |
|  | ***mboat4*** | ***Postprandial*** | 1.7 | 1.3 | 1.7 | 1.6 | 1.4 | 1.7 |
|  |  | ***Feeding*** | 1.6 | 1.6 | 1.9 | 1.6 | 1.3 | 1.7 |
| **GLMM (<P),**  **Status** | ***ghrl1*** |  | ns | ns | ns | ns | ns | ns |
|  | ***ghrl2*** |  | ns | ns | ns | ns | ns | ns |
|  | ***mboat4*** |  | ns | ns | ns | ns | ns | ns |

**TABLE S9** Day 21 and Day 56 midgut mRNA absolute gene expression of *pyya1, pyya2*, *pyyb1*, *pyyb2* and *gcga* in Atlantic salmon fed diets with inclusion of cod side-stream-products, feeding and postprandial fish. Mean ± stdev; n=3. Statistical analysis by generalized linear mixed model (GLMM) assuming a gamma distribution as a function of diet, nutritional status and sampling, followed by Tukey post hoc pairwise tests. *P*<0.05. ns, non-significant.

| **Sampling** | **Status** | **Genes** | **FM10** | **FM-hbg** | **FM-hb** | **FPH-hb** | **FPC-g** | **FM5** | **GLMM (<P),**  **Diet** |
| --- | --- | --- | --- | --- | --- | --- | --- | --- | --- |
| **Day 21** | ***Postprandial*** | ***pyya1*** | 3559 | 3435 | 4245 | 3643 | n.a. | 3741 | ns |
|  |  | ***pyya2*** | 7659 | 7189 | 8822 | 7733 | n.a. | 7149 | ns |
|  |  | ***pyyb1*** | 762 | 625 | 741 | 718 | n.a. | 652 | ns |
|  |  | ***pyyb2*** | 1648 | 1333 | 1823 | 1450 | n.a. | 1418 | ns |
|  |  | ***gcga*** | 5534 | 5553 | 6767 | 5549 | n.a. | 5306 | ns |
|  | ***Feeding*** | ***pyya1*** | 3596 | 3851 | 3679 | 4183 | n.a. | 4310 | ns |
|  |  | ***pyya2*** | 7643 | 8186 | 7597 | 8543 | n.a. | 9478 | ns |
|  |  | ***pyyb1*** | 683 | 697 | 709 | 805 | n.a. | 758 | ns |
|  |  | ***pyyb2*** | 1470 | 1574 | 1263 | 1726 | n.a. | 1605 | ns |
|  |  | ***gcga*** | 5730 | 6625 | 5817 | 6481 | n.a. | 6692 | ns |

| **Sampling** | **Genes** | **Status** | **FM10** | **FM-hbg** | **FM-hb** | **FPH-hb** | **FPC-g** | **FM5** |
| --- | --- | --- | --- | --- | --- | --- | --- | --- |
| **Day 21** | ***pyya1*** | ***Postprandial*** | 3559 | 3435 | 4245 | 3643 | n.a. | 3741 |
|  |  | ***Feeding*** | 3596 | 3851 | 3679 | 4183 | n.a. | 4310 |
|  | ***pyya2*** | ***Postprandial*** | 7659 | 7189 | 8822 | 7733 | n.a. | 7149^x^ |
|  |  | ***Feeding*** | 7643 | 8186 | 7597 | 8543 | n.a. | 9478^y^ |
|  | ***pyyb1*** | ***Postprandial*** | 762 | 625 | 741 | 718 | n.a. | 652 |
|  |  | ***Feeding*** | 683 | 697 | 709 | 805 | n.a. | 758 |
|  | ***pyyb2*** | ***Postprandial*** | 1648 | 1333 | 1823^y^ | 1450 | n.a. | 1418 |
|  |  | ***Feeding*** | 1470 | 1574 | 1263^x^ | 1726 | n.a. | 1605 |
|  | ***gcga*** | ***Postprandial*** | 5534 | 5553^*^ | 6767 | 5549 | n.a. | 5306^x^ |
|  |  | ***Feeding*** | 5730 | 6625^*^ | 5817 | 6481 | n.a. | 6692^y^ |
| **GLMM (<P),**  **Status** | ***pyya1*** |  | ns | ns | ns | ns |  | ns |
|  | ***pyya2*** |  | ns | ns | ns | ns |  | 0.0153 |
|  | ***pyyb1*** |  | ns | ns | ns | ns |  | ns |
|  | ***pyyb2*** |  | ns | ns | 0.0353 | ns |  | ns |
|  | ***gcga*** |  | ns | ^*^trend | ns | ns |  | 0.0260 |

| **Sampling** | **Status** | **Genes** | **FM10** | **FM-hbg** | **FM-hb** | **FPH-hb** | **FPC-g** | **FM5** | **GLMM (<P),**  **Diet** |
| --- | --- | --- | --- | --- | --- | --- | --- | --- | --- |
| **Day 56** | ***Postprandial*** | ***pyya1*** | 4506 | 3788 | 4403 | 4384 | n.a. | 4166 | ns |
|  |  | ***pyya2*** | 7117 | 6762 | 8086 | 7613 | n.a. | 7439 | ns |
|  |  | ***pyyb1*** | 1069 | 879^a^ | 1376^b^ | 1358^b^ | n.a. | 1136 | 0.0291 |
|  |  | ***pyyb2*** | 2067^a^ | 1779^a,*^ | 3381^b^ | 2779^*^ | n.a. | 2212 | 0.0299 / ^*^trend |
|  |  | ***gcga*** | 8692 | 7184 | 7117 | 8395 | n.a. | 7890 | ns |
|  | ***Feeding*** | ***pyya1*** | 5265 | 4390 | 4546 | 4785 | n.a. | 4159 | ns |
|  |  | ***pyya2*** | 8697 | 7686 | 8048 | 8641 | n.a. | 6906 | ns |
|  |  | ***pyyb1*** | 1414^c^ | 860^a^ | 1083 | 1369^bc^ | n.a. | 797^a^ | 0.0159 |
|  |  | ***pyyb2*** | 2931 | 2356 | 2475 | 3003 | n.a. | 2664 | ns |
|  |  | ***gcga*** | 11087^b^ | 8174^a^ | 8770 | 9139 | n.a. | 7565^a^ | 0.0165 |

| **Sampling** | **Genes** | **Status** | **FM10** | **FM-hbg** | **FM-hb** | **FPH-hb** | **FPC-g** | **FM5** |
| --- | --- | --- | --- | --- | --- | --- | --- | --- |
| **Day 56** | ***pyya1*** | ***Postprandial*** | 4506 | 3788 | 4403 | 4384 | n.a. | 4166 |
|  |  | ***Feeding*** | 5265 | 4390 | 4546 | 4785 | n.a. | 4159 |
|  | ***pyya2*** | ***Postprandial*** | 7117^*^ | 6762 | 8086 | 7613 | n.a. | 7439 |
|  |  | ***Feeding*** | 8697^*^ | 7686 | 8048 | 8641 | n.a. | 6906 |
|  | ***pyyb1*** | ***Postprandial*** | 1069^*^ | 879 | 1376 | 1358 | n.a. | 1136^y^ |
|  |  | ***Feeding*** | 1414^*^ | 860 | 1083 | 1369 | n.a. | 797^x^ |
|  | ***pyyb2*** | ***Postprandial*** | 2067^x^ | 1779^*^ | 3381^*^ | 2779 | n.a. | 2212 |
|  |  | ***Feeding*** | 2931^y^ | 2356^*^ | 2475^*^ | 3003 | n.a. | 2664 |
|  | ***gcga*** | ***Postprandial*** | 8692^x^ | 7184 | 7117^*^ | 8395 | n.a. | 7890 |
|  |  | ***Feeding*** | 11087^y^ | 8174 | 8770^*^ | 9139 | n.a. | 7565 |
| **GLMM (<P),**  **Status** | ***pyya1*** |  | ns | ns | ns | ns |  | ns |
|  | ***pyya2*** |  | ^*^trend | ns | ns | ns |  | ns |
|  | ***pyyb1*** |  | ^*^trend | ns | ns | ns |  | 0.0257 |
|  | ***pyyb2*** |  | 0.0453 | ^*^trend | ^*^trend | ns |  | ns |
|  | ***gcga*** |  | 0.0133 | ns | ^*^trend | ns |  | ns |

**TABL S10** Day 21 and Day 56 hindgut mRNA absolute gene expression of *pyya1, pyya2*, *pyyb1*, *pyyb2* and *gcga* in Atlantic salmon fed diets with inclusion of cod side-stream-products, feeding and postprandial fish. Mean ± stdev; n=3. Statistical analysis by generalized linear mixed model (GLMM) assuming a gamma distribution as a function of diet, nutritional status and sampling, followed by Tukey post hoc pairwise tests. *P*<0.05. ns, non-significant.

| **Sampling** | **Status** | **Genes** | **FM10** | **FM-hbg** | **FM-hb** | **FPH-hb** | **FPC-g** | **FM5** | **GLMM (<P),**  **Diet** |
| --- | --- | --- | --- | --- | --- | --- | --- | --- | --- |
| **Day 21** | ***Postprandial*** | ***pyya1*** | low | low | low | low | n.a. | low | ns |
|  |  | ***pyya2*** | 194^a^ | 228 | 298^b^ | 293^b^ | n.a. | 311^b^ | 0.0337 |
|  |  | ***pyyb1*** | 2.6 | 2.1 | 2.5 | 2.8 | n.a. | 2.8 | ns |
|  |  | ***pyyb2*** | 8.0 | 7.3 | 8.6 | 7.7 | n.a. | 8.7 | ns |
|  |  | ***gcga*** | 2019 | 2163 | 2000 | 2333 | n.a. | 2345 | ns |
|  | ***Feeding*** | ***pyya1*** | low | low | low | low | n.a. | low | ns |
|  |  | ***pyya2*** | 212 | 262 | 210 | 211 | n.a. | 222 | ns |
|  |  | ***pyyb1*** | 2.4 | 2.6 | 2.6 | 2.7 | n.a. | 3.2 | ns |
|  |  | ***pyyb2*** | 9.8 | 8.1 | 8.1 | 7.5 | n.a. | 8.2 | ns |
|  |  | ***gcga*** | 2061 | 2264 | 2035 | 1840 | n.a. | 2054 | ns |

| **Sampling** | **Genes** | **Status** | **FM10** | **FM-hbg** | **FM-hb** | **FPH-hb** | **FPC-g** | **FM5** |
| --- | --- | --- | --- | --- | --- | --- | --- | --- |
| **Day 21** | ***pyya1*** | ***Postprandial*** | low | low | low | low | n.a. | low |
|  |  | ***Feeding*** | low | low | low | low | n.a. | low |
|  | ***pyya2*** | ***Postprandial*** | 194 | 228 | 298^y^ | 293^y^ | n.a. | 311^y^ |
|  |  | ***Feeding*** | 212 | 262 | 210^x^ | 211^x^ | n.a. | 222^x^ |
|  | ***pyyb1*** | ***Postprandial*** | 2.6 | 2.1 | 2.5 | 2.8 | n.a. | 2.8 |
|  |  | ***Feeding*** | 2.4 | 2.6 | 2.6 | 2.7 | n.a. | 3.2 |
|  | ***pyyb2*** | ***Postprandial*** | 8.0 | 7.3 | 8.6 | 7.7 | n.a. | 8.7 |
|  |  | ***Feeding*** | 9.8 | 8.1 | 8.1 | 7.5 | n.a. | 8.2 |
|  | ***gcga*** | ***Postprandial*** | 2019 | 2163 | 2000 | 2333^*^ | n.a. | 2345 |
|  |  | ***Feeding*** | 2061 | 2264 | 2035 | 1840^*^ | n.a. | 2054 |
| **GLMM (<P),**  **Status** | ***pyya1*** |  | ns | ns | ns | ns |  | ns |
|  | ***pyya2*** |  | ns | ns | 0.0126 | 0.0236 |  | 0.0162 |
|  | ***pyyb1*** |  | ns | ns | ns | ns |  | ns |
|  | ***pyyb2*** |  | ns | ns | ns | ns |  | ns |
|  | ***gcga*** |  | ns | ns | ns | ^*^trend |  | ns |

| **Sampling** | **Status** | **Genes** | **FM10** | **FM-hbg** | **FM-hb** | **FPH-hb** | **FPC-g** | **FM5** | **GLMM (<P),**  **Diet** |
| --- | --- | --- | --- | --- | --- | --- | --- | --- | --- |
| **Day 56** | ***Postprandial*** | ***pyya1*** | low | low | low | low | n.a. | low | ns |
|  |  | ***pyya2*** | 165 | 153 | 163 | 196 | n.a. | 190 | ns |
|  |  | ***pyyb1*** | 1.4 | 1.8^*^ | 1.3^*^ | 1.6 | n.a. | 1.7 | trend |
|  |  | ***pyyb2*** | 7.6 | 8.2 | 8.7 | 8.6 | n.a. | 8.5 | ns |
|  |  | ***gcga*** | 2206 | 1806^a^ | 2365 | 2748^b^ | n.a. | 2404 | 0.0187 |
|  | ***Feeding*** | ***pyya1*** | low | Low | low | Low | n.a. | low | ns |
|  |  | ***pyya2*** | 186 | 202^*^ | 188 | 248^b^ | n.a. | 142^a,*^ | 0.0009 / ^*^trend |
|  |  | ***pyyb1*** | 2.0 | 2.1 | 2.0 | 2.0 | n.a. | 2.0 | ns |
|  |  | ***pyyb2*** | 8.5 | 9.2 | 9.6 | 10.9 | n.a. | 11.0 | ns |
|  |  | ***gcga*** | 2546 | 2167 | 2462 | 2030 | n.a. | 2191 | ns |

| **Sampling** | **Genes** | **Status** | **FM10** | **FM-hbg** | **FM-hb** | **FPH-hb** | **FPC-g** | **FM5** |
| --- | --- | --- | --- | --- | --- | --- | --- | --- |
| **Day 56** | ***pyya1*** | ***Postprandial*** | low | low | low | low | n.a. | low |
|  |  | ***Feeding*** | low | low | low | low | n.a. | low |
|  | ***pyya2*** | ***Postprandial*** | 165 | 153^x^ | 163 | 196 | n.a. | 190^y^ |
|  |  | ***Feeding*** | 186 | 202^y^ | 188 | 248 | n.a. | 142^x^ |
|  | ***pyyb1*** | ***Postprandial*** | 1.4^x^ | 1.8 | 1.3^x^ | 1.6 | n.a. | 1.7 |
|  |  | ***Feeding*** | 2.0^y^ | 2.1 | 2.0^y^ | 2.0 | n.a. | 2.0 |
|  | ***pyyb2*** | ***Postprandial*** | 7.6 | 8.2 | 8.7 | 8.6^*^ | n.a. | 8.5^*^ |
|  |  | ***Feeding*** | 8.5 | 9.2 | 9.6 | 10.9^*^ | n.a. | 11.0^*^ |
|  | ***gcga*** | ***Postprandial*** | 2206 | 1806 | 2365 | 2748^y^ | n.a. | 2404 |
|  |  | ***Feeding*** | 2546 | 2167 | 2462 | 2030^x^ | n.a. | 2191 |
| **GLMM (<P),**  **Status** | ***pyya1*** |  | ns | ns | ns | ns |  | ns |
|  | ***pyya2*** |  | ns | 0.0451 | ns | ns |  | 0.0347 |
|  | ***pyyb1*** |  | 0.0313 | ns | 0.0018 | ns |  | ns |
|  | ***pyyb2*** |  | ns | ns | ns | ^*^trend |  | ^*^trend |
|  | ***gcga*** |  | ns | ns | ns | 0.0271 |  | ns |

**
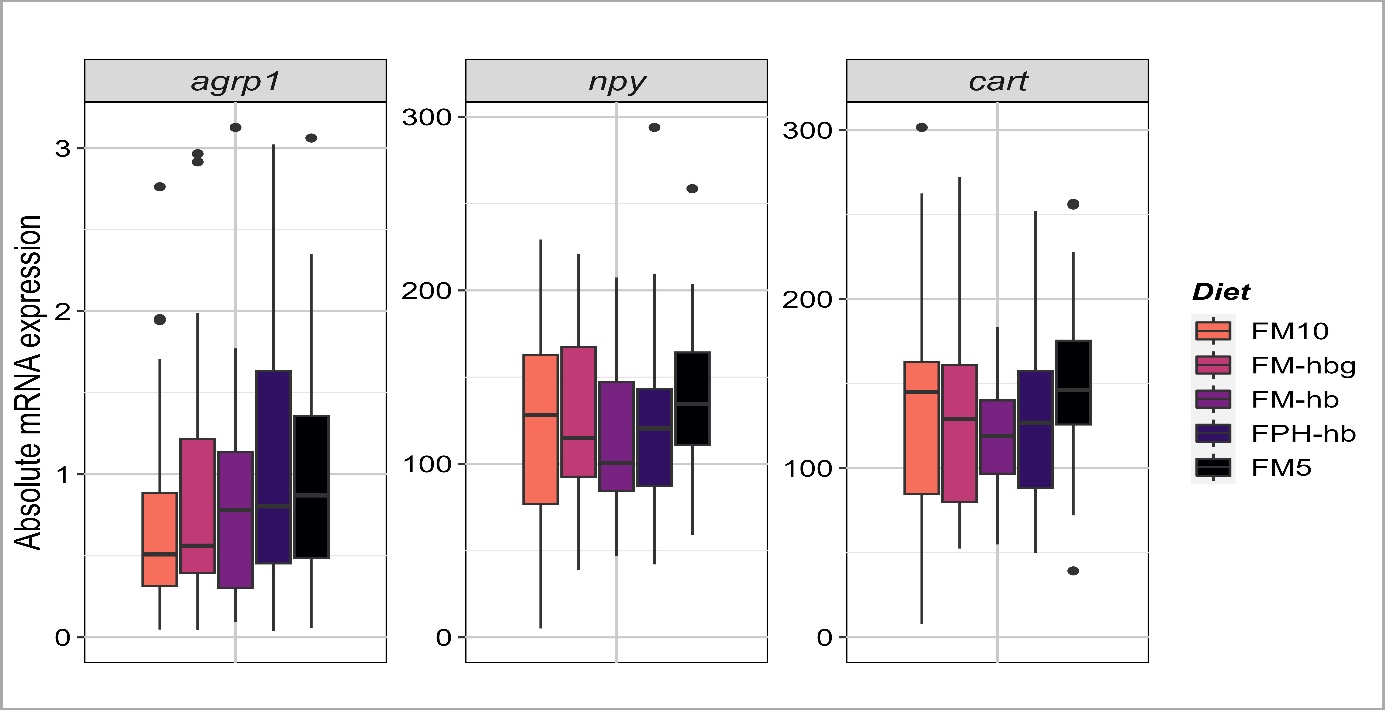
**

**FIGURE S3** Comparison of the absolute mRNA expression in the brain (*agrp1, npy, cart*) dependent on diet, and independent of status or sampling time. Boxplot shows the median (solid horizontal line), the first and third quartiles (lower and upper limit of the box), the whiskers indicate the 1.5 * interquartile range, and dots are data points outside of the defined range.

**
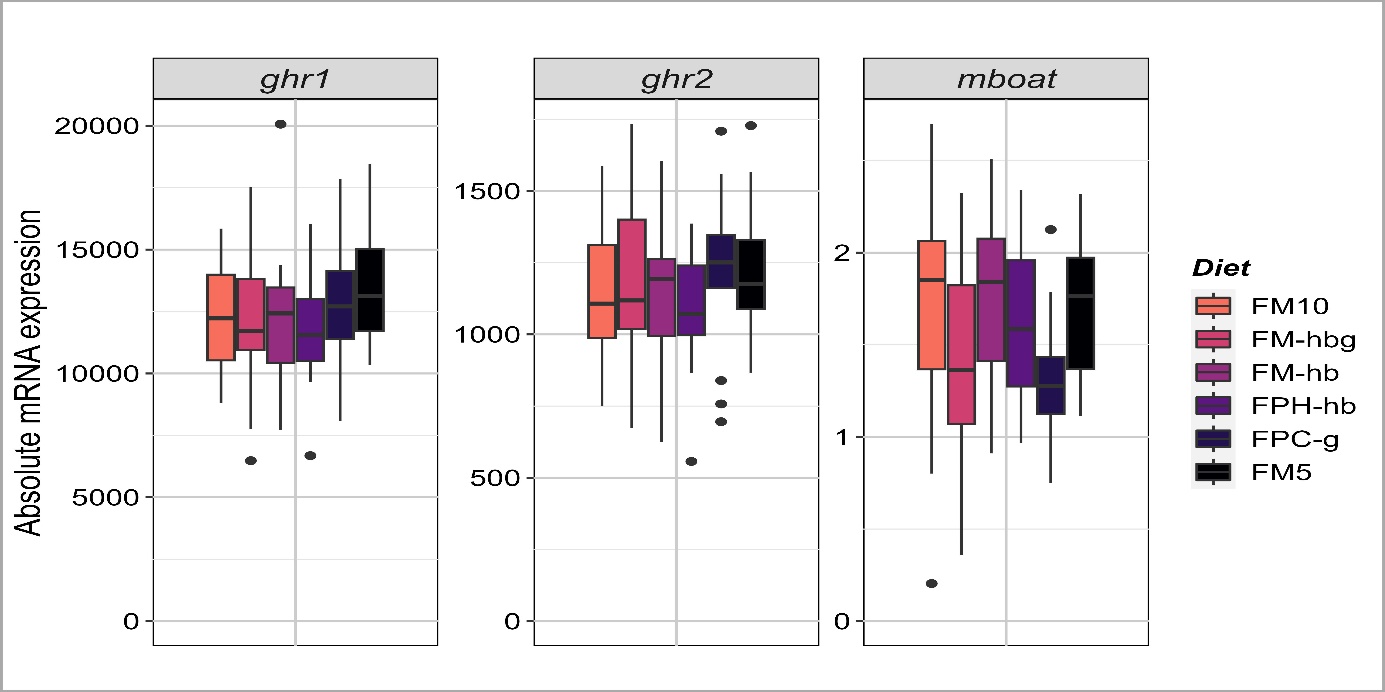
 FIGURE S4** Comparison of the absolute mRNA expression in stomach (*ghrl1, ghrl2, mboat4*) dependent on diet, and independent on status or sampling time. Boxplot shows the median (solid horizontal line), the first and third quartiles (lower and upper limit of the box), the whiskers indicate the 1.5 * interquartile range, and dots are data points outside of the defined range.

**
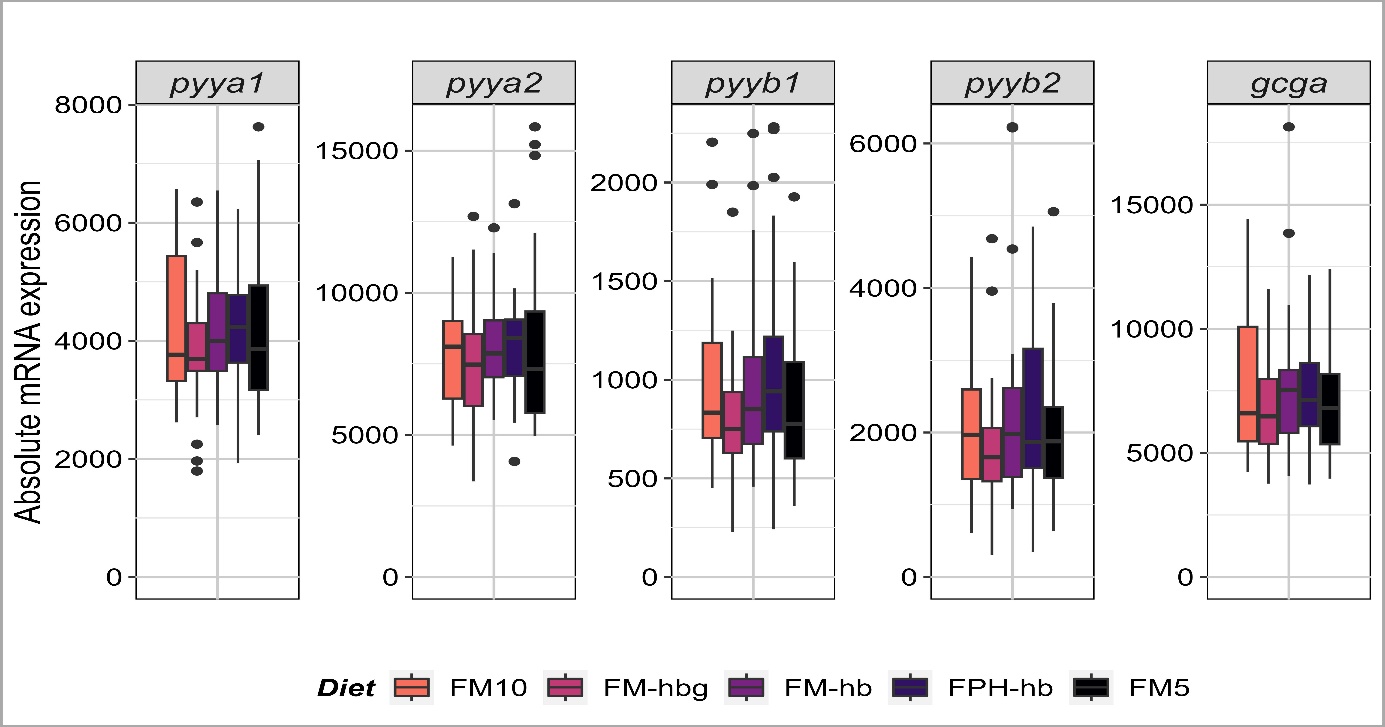
 FIGURE S5** Comparison of the absolute mRNA expression in midgut (*pyya1, pyya2, pyyb1, pyyb2, gcga*) dependent on diet, and independent on status or sampling time. Boxplot shows the median (solid horizontal line), the first and third quartiles (lower and upper limit of the box), the whiskers indicate the 1.5 * interquartile range, and dots are data points outside of the defined range.

**
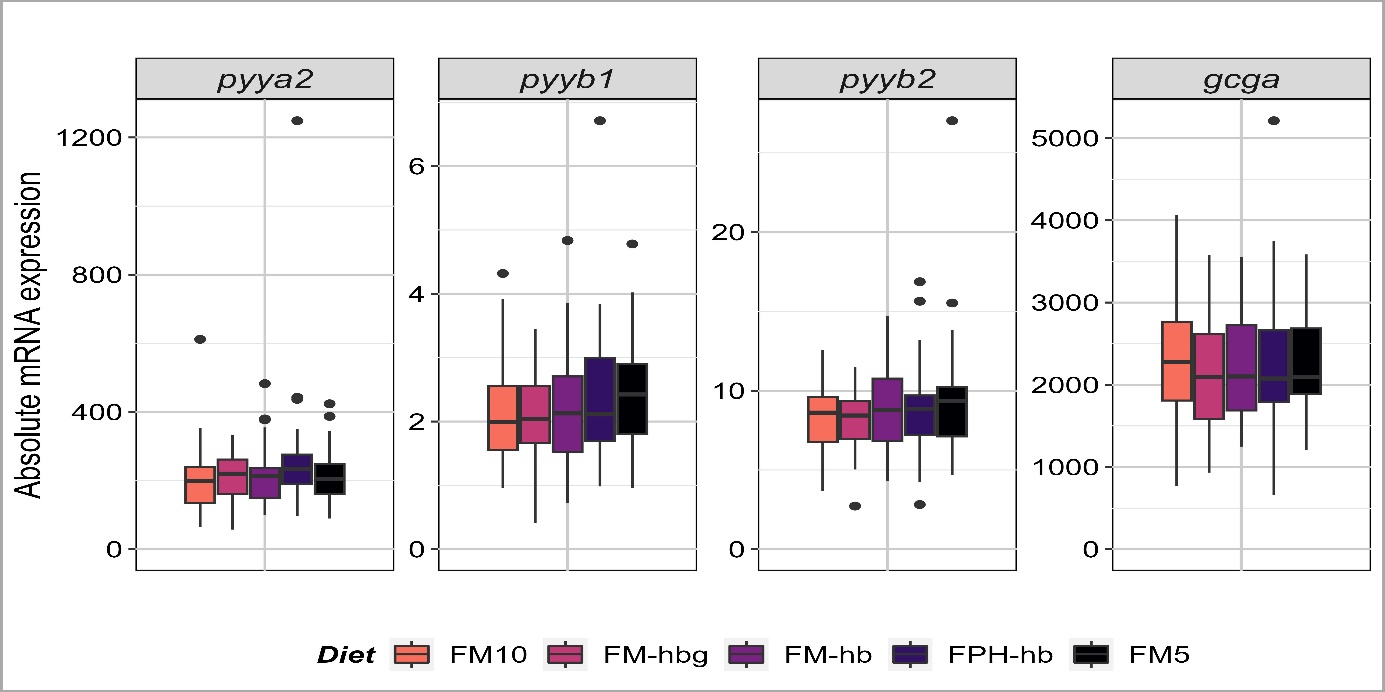
 FIGURE S6** Comparison of the absolute mRNA expression in hindgut (*pyya1, pyya2, pyyb1, pyyb2, gcga*) dependent on diet, and independent on status or sampling time. Boxplot shows the median (solid horizontal line), the first and third quartiles (lower and upper limit of the box), the whiskers indicate the 1.5 * interquartile range, and dots are data points outside of the defined range. For *pyya1*: Unreliable data due to trace expression levels.

**FIGURE S7** Random effect of Tank in brain gene expression models
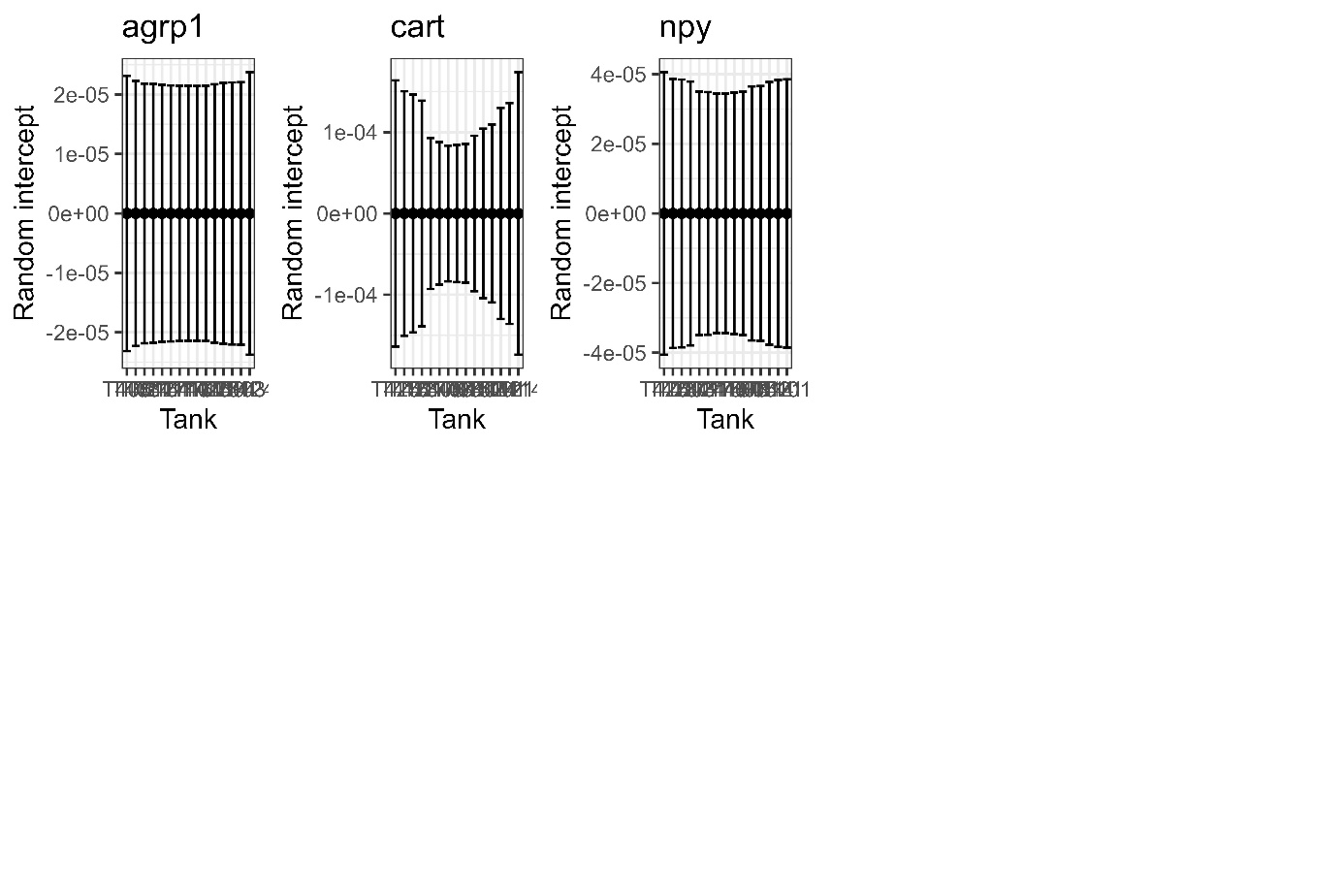


**FIGURE S8** Random effect of Tank in stomach gene expression models


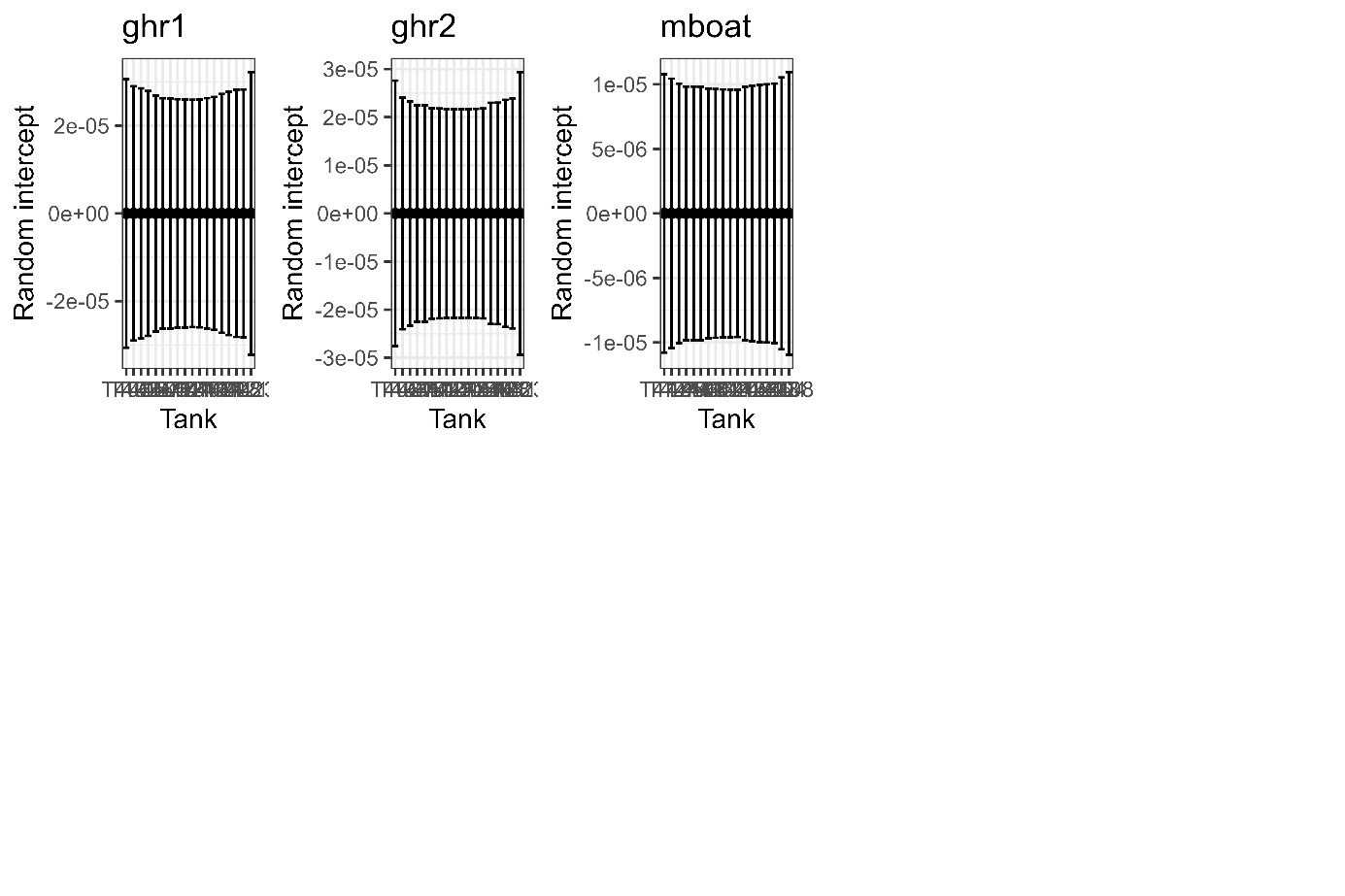


**FIGURE S9** Random effect of Tank in midgut gene expression models**
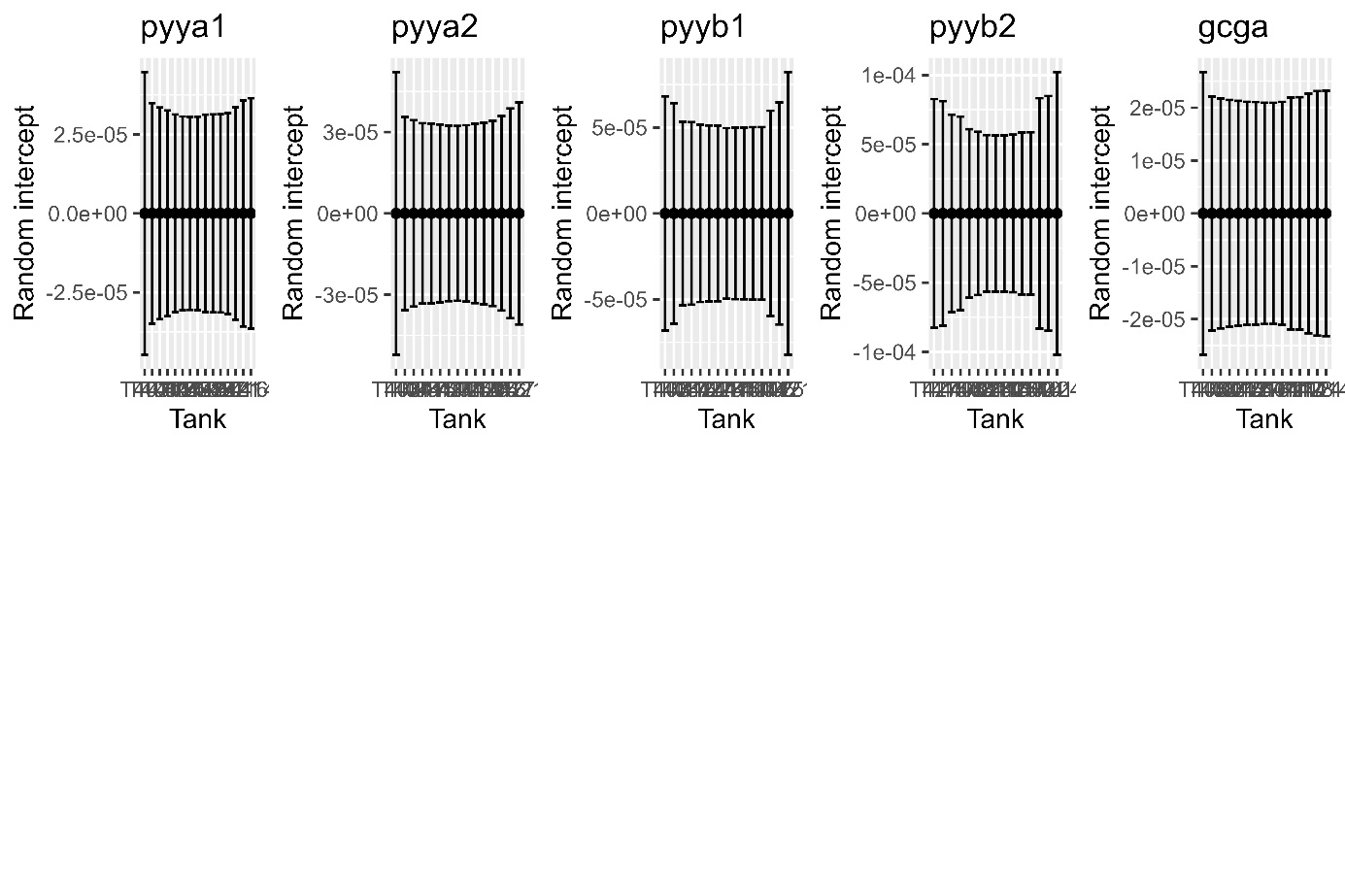
**

**FIGURE S10** Random effect of Tank in hindgut gene expression models
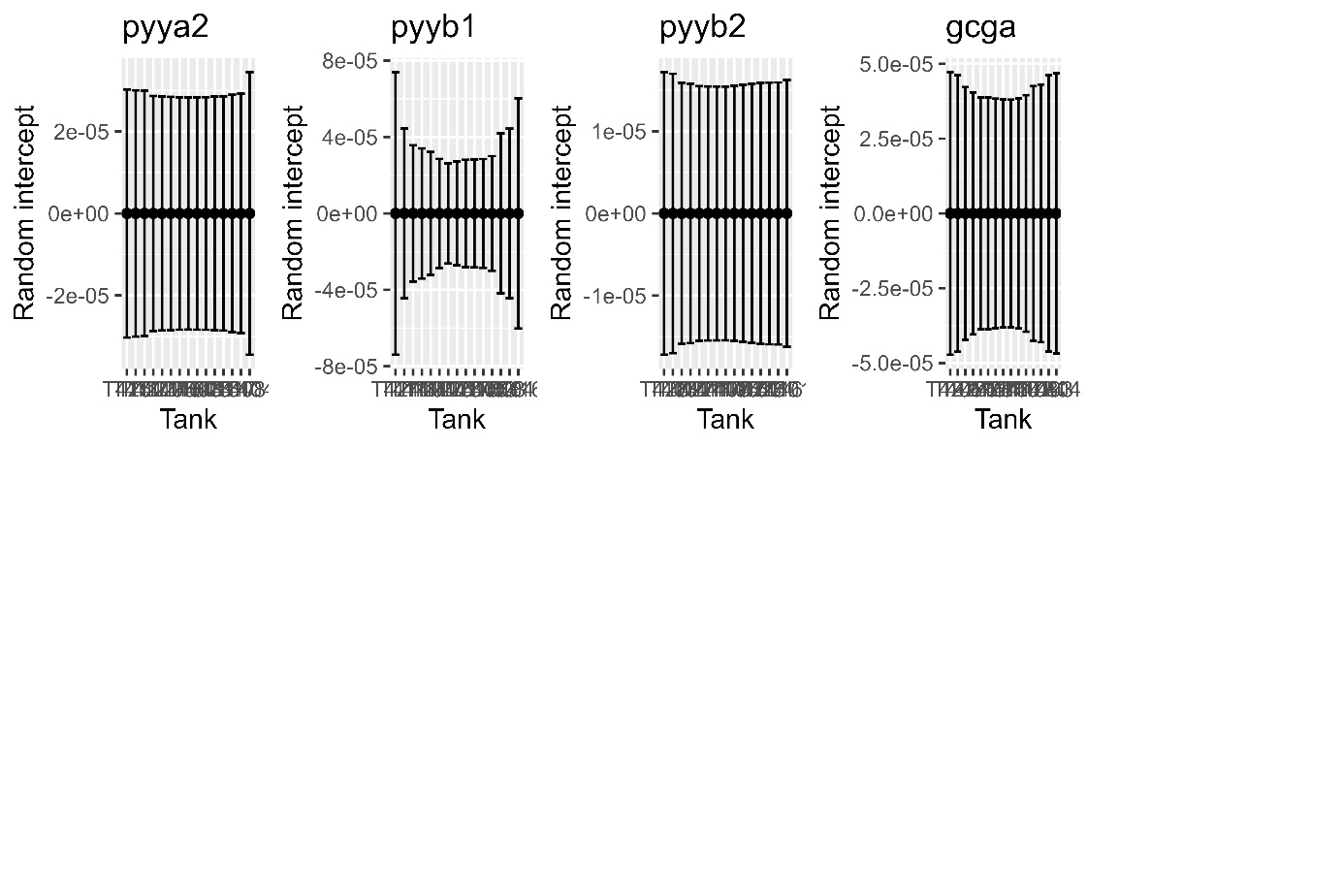

Supplement: Supporting Information — Extensive analysis of the ingredient composition of AAs, FAA, nucleotides, FA, and lipid classes is presented in Tables S1 and S2. Extensive analysis of the experimental feed composition of AAs, FAA, FA, and lipid classes is presented in Tables S3 and S4. Micro-CT pore structure and distribution analysis of the experimental feeds are presented in Figures S1 and S2. Atlantic salmon ADC values for AAs are presented in Tables S5. Atlantic salmon biometrics from Day 21 is presented in Tables S6. Extensive statistical analysis of gene expression from whole brain, stomach, midgut, and hindgut are presented in Tables S7–S10, respectively, and overall expression patterns in Figures S3–S6, respectively. Random effect of tank in gene expression models are presented in Figures S7–S10, respectively, for whole brain, stomach, midgut, and hindgut. [file 4872889.f1.docx]
